# Chemosensory detection of polyamine metabolites guides *C. elegans* to nutritive microbes

**DOI:** 10.1126/sciadv.adj4387

**Published:** 2024-03-22

**Authors:** Benjamin Brissette, Lia Ficaro, Chenguang Li, Drew R. Jones, Sharad Ramanathan, Niels Ringstad

**Affiliations:** ^1^ Department of Cell Biology, Neuroscience and Physiology, Neuroscience Institute, NYU School of Medicine, New York, NY 10016, USA.; ^2^ Department of Biochemistry and Pharmacology, NYU School of Medicine, New York, NY 10016, USA.; ^3^ Biophysics Program, Harvard University, Cambridge, MA 02138, USA.; ^4^ Department of Molecular and Cellular Biology, Harvard University, Cambridge, MA 02138, USA.; ^5^ John A. Paulson School of Engineering and Applied Sciences, Harvard University, Cambridge, MA 02138, USA.

## Abstract

Much is known about molecular mechanisms by which animals detect pathogenic microbes, but how animals sense beneficial microbes remains poorly understood. The roundworm *Caenorhabditis elegans* is a microbivore that must distinguish nutritive microbes from pathogens. We characterized a neural circuit used by *C. elegans* to rapidly discriminate between nutritive bacteria and pathogens. Distinct sensory neuron populations responded to chemical cues from nutritive *Escherichia coli* and pathogenic *Enterococcus faecalis*, and these neural signals are decoded by downstream AIB interneurons. The polyamine metabolites cadaverine, putrescine, and spermidine produced by *E. coli* activate this neural circuit and elicit positive chemotaxis. Our study shows how polyamine odorants can be sensed by animals as proxies for microbe identity and suggests that, hence, polyamines might have widespread roles brokering host-microbe interactions.

## Teaser

Polyamines can be sensed as a proxy for microbe identity and might have widespread roles brokering host-microbe interactions.

## INTRODUCTION

Microbes provide many benefits to their animal hosts. In the gut, microbes perform biosynthetic functions that complement metabolic shortcomings of their hosts (*1*). At epithelial barriers, microbes serve critical immunomodulatory functions by tempering host immunity and systemically damping inflammation (*2*). Commensal microbes also interact with the host nervous system by providing neuroactive metabolites capable of modulating host physiology and exerting strong and specific effects on affect and behavior (*3*). Given the strong influence that microbes have on host fitness, it should follow that animals are endowed with the ability to detect those microbes to preferentially associate with them. Little, however, is known about if and how animals sense microbes that improve fitness.

The free-living nematode *Caenorhabditis elegans* feeds on environmental microbes, which provide caloric energy and fulfill essential biosynthetic functions, for example, by synthesizing heme, cholesterol, and metabolic cofactors (*4*). The diverse microbes found in the natural environment of *C. elegans* vary in their ability to support *C. elegans* growth and fitness (*5*–*7*). Furthermore, *C. elegans* is susceptible to a number of microbial pathogens (*7*, *8*). It is, therefore, critical that *C. elegans* discriminate nutritive microbes that support growth from those microbes that are less nutritive or pathogenic. The nervous system of *C. elegans* plays a major role in mediating interactions with environmental microbes. *C. elegans* learns to recognize and subsequently avoid microbial pathogens by linking microbe-specific chemical cues with infection and sickness (*9*–*12*). *C. elegans* also instinctively avoids some pathogenic microbes and is attracted to other nutritive microbes, indicating that *C. elegans* is endowed with innate behaviors that drive a preference for nonpathogenic microbes (*6*, *9*, *13*, *14*).

Both learned and innate microbe preferences require chemosensory detection of microbe-specific cues. *C. elegans* has a sophisticated chemosensory nervous system capable of encoding a wide array of chemical stimuli (*15*, *16*). To date, only a few microbe-derived chemical signals that instruct *C. elegans* behavior have been identified, mainly in the context of pathogen recognition (*17*–*19*). To better understand how animals recognize microbes, we studied the neural basis of an innate preference of *C. elegans* for nutritive *Escherichia coli* over pathogenic *Enterococcus faecalis*. Here, we report the identification of chemosensory neurons that recognize *E. coli*–enriched metabolites, a key downstream interneuron required for chemotaxis to nutritive *E. coli*, and the discovery of polyamine metabolites that are sensed by this circuit as a signature of nutritive bacteria.

## RESULTS

### Chemosensation rapidly distinguishes pathogenic and nutritive microbes

**Fig. 1.**
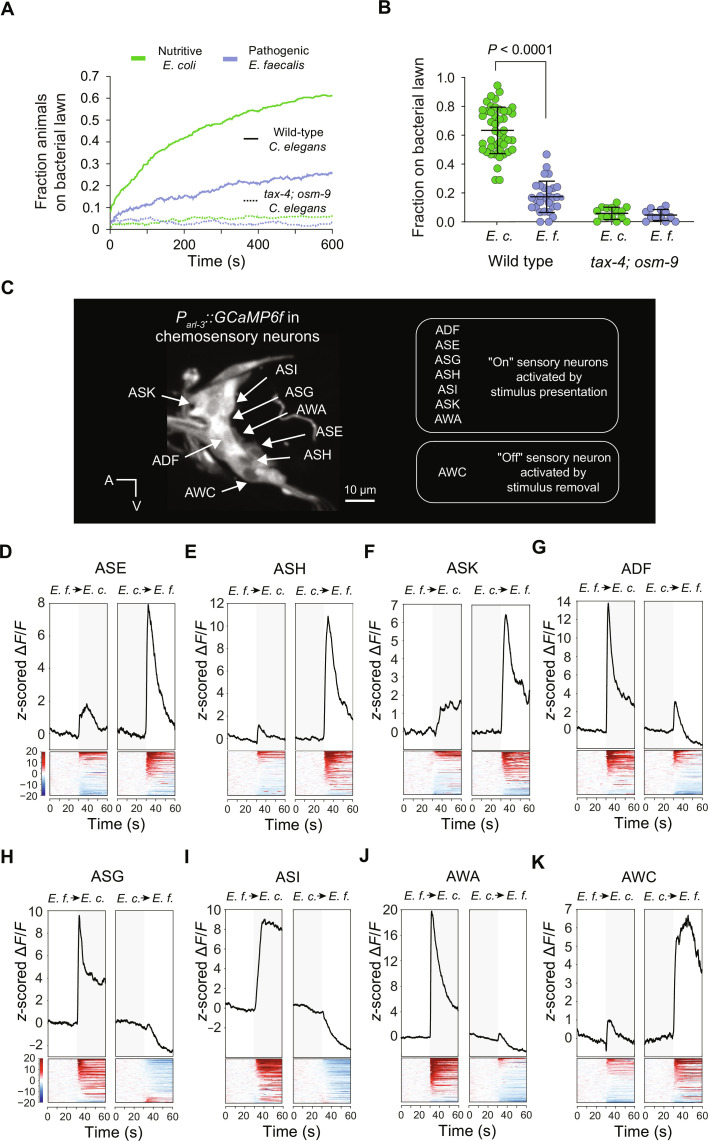
*C. elegans* uses chemosensation to rapidly identify and respond to beneficial microbes. (**A**) Average occupancy over time of *E. coli* (*E. c.*; green) or *E. faecalis* (*E. f.*; blue) lawns by the wild type (solid line) or by chemosensory-defective *tax-4*; *osm-9* double-mutants (dashed line). For the wild type, 45 trials were conducted on *E. coli* and 30 on *E. faecalis*; for *tax-4*; *osm-9* mutants, 15 trials were conducted on *E. coli* and 12 on *E. faecalis.* Thirty animals were used in each trial. (**B**) End-point data for individual occupancy trials. Bars indicate mean ± SD. Student’s *t* test was used to compute the indicated *P* value. Each point represents one trial of 30 animals. (**C**) GCaMP6f is expressed in ciliated sensory neurons using the *arl-3* promoter. Cell bodies of some microbe-responsive neurons are indicated with arrows. (**D** to **F**) *z*-scored calcium signals in *E. faecalis*–responsive ASE, ASH, and ASK neurons in response to switches between *E. faecalis*–conditioned and *E. coli*–conditioned media. Data are plotted from 108 trials of ASEs, 117 trials of ASHs, and 105 trials of ASKs. (**G** to **K**) *z*-scored calcium signals in *E. coli*–responsive ADF, ASG, ASI, AWA, and AWC neurons to switches between *E. faecalis*–conditioned and *E. coli*–conditioned media. Data are plotted from 70 trials of ADFs, 92 trials of ASGs, 86 trials of ASIs, 110 trials of AWAs, and 116 trials of AWCs. Note that AWCs respond to stimulus removal and are therefore grouped with neurons that preferentially respond to *E. coli* presentation. In (D) to (K), traces of the mean *z*-scored signals are shown above heatmaps showing the *z*-scored signals from individual trials.

Some nutritive bacteria are highly attractive and elicit dwelling on bacterial lawns, and some pathogenic bacteria are aversive and promote avoidance and roaming (*13*, *20*). We compared the behavior of well-fed *C. elegans* hermaphrodites in an arena with nutritive *E. coli* to that of animals in an arena with *E. faecalis*, a pathogenic microbe previously shown to be innately aversive to *C. elegans* (*13*). Video tracking showed that animals rapidly accumulated on patches of nutritive *E. coli*, whereas patches of pathogenic *E. faecalis* failed to attract and retain animals (Fig. 1, A and B). This difference in behavior appeared within minutes (Fig. 1A), suggesting that sensation of bacteria, not infection, instructs these distinct microbe-response behaviors. Consistent with this hypothesis, mutation of *tax-4* and *osm-9*, which encode ion channels that are required for chemosensory transduction (*21*, *22*), abrogated dwelling of *C. elegans* on patches of nutritive bacteria (Fig. 1, A and B).

To determine how *C. elegans* senses nutritive and pathogenic bacteria, we generated a transgenic animal expressing the high-sensitivity calcium indicator GCaMP6f (*23*) in all sensory neurons (Fig. 1C). We recorded optical signals from sensory neurons of animals restrained in a microfluidic device (*24*) that allowed us to sequentially present media conditioned either by nutritive *E. coli* or by pathogenic *E. faecalis*. This protocol was designed to first adapt sensory neurons to media conditioned by one microbe and then determine whether media conditioned by another microbe contained distinct metabolites that could be detected by *C. elegans*. We observed that *C. elegans* sensory neurons robustly reported differences between *E. coli*– and *E. faecalis*–conditioned media. ASE, ASH, and ASK neurons preferentially responded to presentation of media conditioned by *E. faecalis*, indicating that these neurons detect pathogen-specific metabolites (Fig. 1, D to F). By contrast, ADF, ASG, ASI, and AWA neurons preferentially responded to presentation of media conditioned by *E. coli* (Fig. 1, G to J), indicating that these neurons detect metabolites specific to nutritive *E. coli*. Whereas most *C. elegans* sensory neurons respond to stimulus presentation, AWC neurons are activated by removal of the odorants that they detect (*25*). We observed robust responses of AWC neurons to withdrawal of *E. coli*–conditioned media (Fig. 1K), and therefore, we grouped AWCs together with ADF, ASG, ASI, and AWA neurons as chemosensory neurons that preferentially respond to chemical cues from nutritive *E. coli*. AWB sensory neurons responded both to cues specific to *E. coli* and cues specific to *E. faecalis*, while ADL and ASJ neurons displayed little or no response to transitions from one conditioned medium to the other (fig. S1).

The microbe-responsive neuron types that we identified are present as bilaterally symmetric pairs of neurons (*26*). In the case of ASE and AWC neurons, left and right homologs are functionally distinct and can respond to different stimuli (*27*, *28*). We did not, however, find evidence of lateralized functions for ASE neurons in microbe sensing (fig. S2). Also, more than 70% of AWCs responded to *E. coli*–conditioned media (Fig. 1K), which is significantly higher than the expected rate if only one AWC neuron was tuned to detect *E. coli*. We observed that in many instances, responses to microbe-conditioned media seemed more variable than those reported from prior studies of sensory responses to monomolecular odorants and gustants (*15*). The observed variability might be a consequence of multiple odorants acting on a sensory neuron, or it might emerge from interactions between the multiple sensory neurons activated by microbe-conditioned media. Together, these data indicated that the chemosensory nervous system of *C. elegans* rapidly encodes microbe identities.

### AIB interneurons decode sensory responses to microbes to guide foraging behavior

**Fig. 2.**
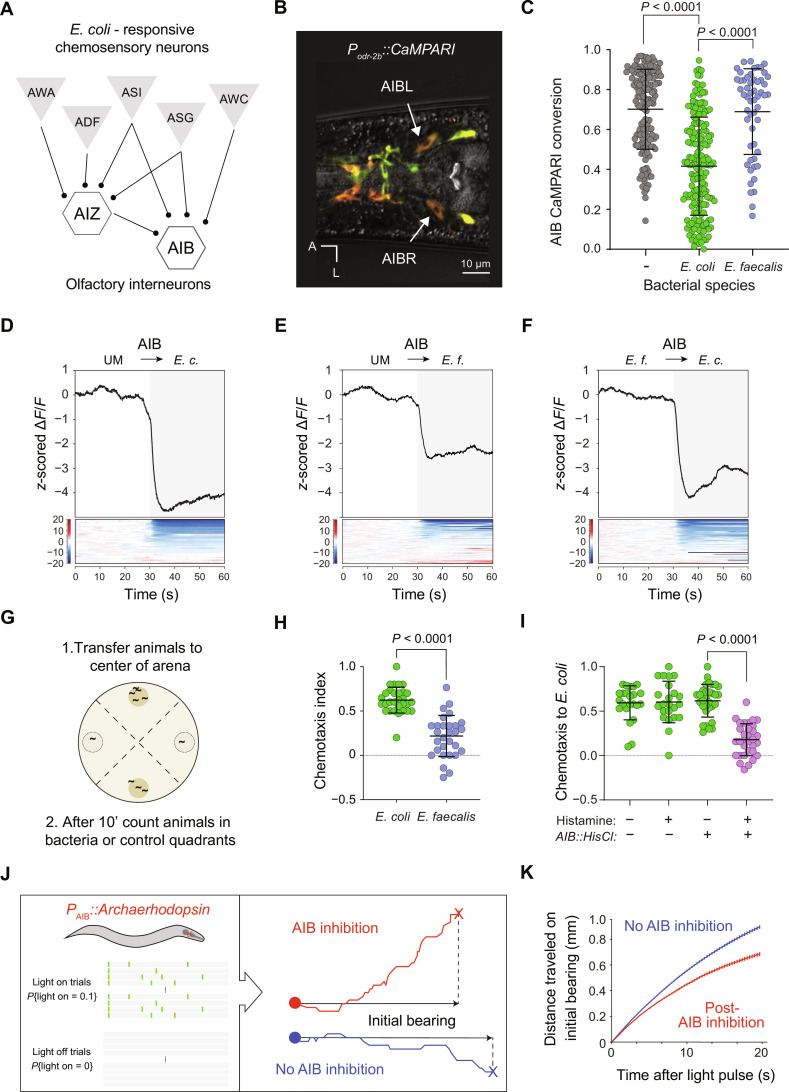
AIB interneurons integrate inputs from chemosensory neurons that detect beneficial microbes. (**A**) *E. coli*–responsive chemosensory neurons provide convergent inputs to AIB and AIZ interneurons. (**B**) CaMPARI expression using *odr-2b* regulatory sequences. A, anterior; L, left. Scale bar, 10 μm. (**C**) CaMPARI signals in control animals (*n* = 150 cells) and animals exposed to *E. coli* (*n* = 171 cells) or *E. faecalis* (*n* = 54 cells). Error bars show mean and SD. *P* values were computed using the Kolmogorov-Smirnov test and corrected for multiple comparisons. Each point represents one cell. (**D** to **F**) Calcium responses of AIB interneurons to *E. coli*–conditioned media measured using GCaMP6f (*n* = 33 trials), *E. faecalis*–conditioned media (*n* = 60 trials), and *E. coli*-conditioned media after adaptation to *E. faecalis*–conditioned media (*n* = 70 trials). (**G**) An assay for rapid chemotaxis of *C. elegans* to microbes. Dashed circles are control quadrants, and filled circles are bacteria quadrants. (**H**) Chemotaxis to patches of *E. coli* and *E. faecalis* (*n* = 30 trials each). Each trial used 20 animals. Chemotaxis index = (animals in bacteria quadrants − animals in control quadrants)/(animals in all quadrants). *P* value was computed using Student’s *t* test. Error bars represent mean and SD. (**I**) Chemotaxis to *E. coli* by the wild type or transgenics expressing HisCl in AIBs. Twenty-three trials of the wild type were performed with and without histamine. Thirty-one trials of transgenics were performed without histamine and 32 with histamine. Each trial used 20 animals. Error bars represent mean and SD. *P* value was computed using Student’s *t* test. Each point represents one trial. (**J**) An assay for determining effects of optogenetic silencing of AIB on foraging. (**K**) Distance traveled in the direction along the initial heading after receiving pulses of AIB inhibition (red, *n* = 8232 “light-on”) or not (blue, *n* = 9608 “light-off”). Error bars show SEM.

How are sensory responses to microbes decoded to instruct foraging behavior? In the *C. elegans* sensory nervous system, a small number of interneurons receive convergent input from distinct sensory neuron types (*26*, *29*). Microbe-responsive chemosensory neurons synapse onto AIB and AIZ interneurons, with AIB interneurons occupying a position as a bottleneck between sensory neurons and premotor neurons and (*30*) (Fig. 2A). Because AIBs receive both excitatory and inhibitory signals from chemosensory neurons (*25*, *31*, *32*), the ensemble of neurons activated by microbe-conditioned media could either activate or inhibit AIBs. To determine how AIB activity is affected by activation of microbe-sensing neurons, we first used the genetically encoded calcium integrator CaMPARI (*33*) to measure how exposure to nutritive or pathogenic microbes affects AIB activity in freely behaving animals. We expressed CaMPARI in a small set of neurons that included AIBs using regulatory sequences from the *odr-2* gene (Fig. 2B) (*34*) and measured calcium-dependent photoconversion of CaMPARI during acute exposure to either *E. coli* or *E. faecalis* using a recently reported protocol (*35*). Compared to control animals in an empty arena, the AIB neurons of animals exposed to nutritive *E. coli* showed markedly reduced CaMPARI photoconversion (Fig. 2C), indicating that sensation of *E. coli* inhibited AIB interneurons. By contrast, exposure to pathogenic *E. faecalis* did not have a marked effect on AIB activity as measured by CaMPARI conversion (Fig. 2C). We observed similar differential effects of *E. coli*– and *E. faecalis*–conditioned media on AIZ neurons expressing CaMPARI (fig. S3A). To determine the dynamics of how AIBs respond to microbe-specific cues, we used the fast calcium indicator GCaMP6f to record AIB calcium signals in restrained animals during exposure to media conditioned either by *E. coli* or *E. faecalis*. Exposure to *E. coli*–conditioned media caused a rapid drop in AIB calcium (Fig. 2D), consistent with the inhibition of AIB by exposure to *E. coli* reported by CaMPARI in freely behaving animals (Fig. 2C). GCaMP revealed that *E. faecalis*–sensing neurons also elicited a decrease in AIB calcium, but inhibitory AIB responses to *E. faecalis* metabolites were much smaller than AIB responses to *E. coli* metabolites (Fig. 2E). Furthermore, when animals were adapted to *E. faecalis*–conditioned medium and then exposed to *E. coli*–conditioned medium, AIBs displayed a large inhibitory response (Fig. 2F), indicating that chemosensory neurons that detect *E. coli*–specific metabolites signal to AIB interneurons, as AIBs in *tax-4*; *osm-9* double mutants did not respond (fig. S3B). These data indicate that AIBs are inhibited by the ensemble of sensory neurons that detect *E. coli*–specific cues and suggest that inhibition of AIBs is important for behavioral responses to this nutritive microbe.

We next tested whether AIBs are required for behaviors elicited by sensation of nutritive *E. coli*. We assayed two behaviors that underlie the rapid accumulation of *C. elegans* on patches of *E. coli* observed by video tracking (Fig. 1A). First, we measured chemotaxis to the bacterial patch (Fig. 2G). Patches of *E. coli* were significantly more attractive to animals than were patches of *E. faecalis* (Fig. 2H). Second, we measured the probability of leaving a patch of bacteria after entry (fig. S4A). We found that animals rarely left patches of nutritive *E. coli*, whereas they frequently left patches of pathogenic *E. faecalis* (fig. S4B). We next tested whether AIBs were required for either of these microbe-response behaviors. Chemogenetic silencing of AIBs using the histamine-gated chloride channel HisCl (*36*) had little or no effect on the probability of leaving patches of *E. coli* (fig. S4C) but strongly reduced chemotaxis to those patches (Fig. 2I). These data indicate that chemosensory input to AIB interneurons is required for efficient navigation to a source of nutritive bacteria, but that a distinct neural mechanism promotes dwelling on bacterial patches.

To determine the role of AIBs in chemotaxis toward nutritive bacteria, we measured how optogenetic inhibition of AIBs affects *C. elegans* locomotion. We exposed foraging animals expressing Archaerhodopsin in AIBs to randomly timed 1-s pulses of light and compared the trajectories of animals that had not received AIB inhibition to the trajectories of those that had (Fig. 2J). This experimental design permitted comparisons of behavior during inhibition to behavior in the absence of inhibition within a single animal. We observed no significant effect of AIB inhibition on speed over 20 s (fig. S5); however, animals that experienced inhibition of AIBs changed their bearing such that they traveled shorter distances along their initial heading during that period (Fig. 2K). This observation is consistent with prior studies that used cell ablation and chemogenetic inhibition to show a role for AIB in controlling reorientations during foraging (*36*–*38*). These data support a model in which AIB mediates reorientations after inhibition is removed. Because chemical cues from nutritive *E. coli* cause acute inhibition of AIBs, these data predict that animals will change bearing when they experience a decrease in the concentration of *E. coli*–derived cues and suggest a role for AIBs in canonical mechanisms of chemotaxis, e.g., biased random walks and klinotaxis (*39*–*42*). Our data suggest that the extent of inhibition in AIB neurons may compute how attractive or aversive chemosensory stimuli are.

### *E. coli* polyamine metabolites activate microbe-sensing neurons and guide chemotaxis

**Fig. 3.**
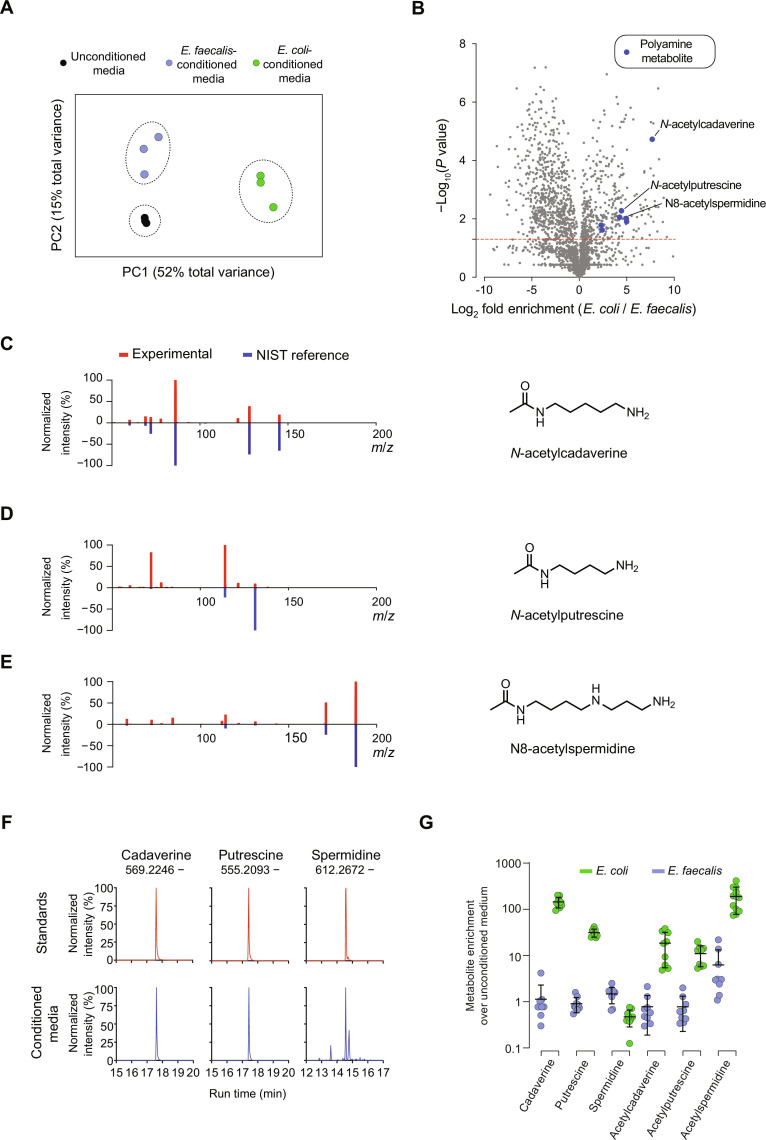
Polyamine metabolites are markers of nutritive *E. coli*. (**A**) Principal components analysis of the metabolomes of unconditioned medium, *E. coli*–conditioned medium, and *E. faecalis*–conditioned medium. Three replicates of each condition were analyzed. (**B**) Volcano plot comparing *E. coli*– and *E. faecalis*–conditioned media. Log_2_ fold enrichment in *E. coli* over *E. faecalis* is plotted on the horizontal axis, and the associated *P* value is plotted on the vertical axis. The red dashed line shows *P* = 0.05. Polyamine metabolites are highlighted in blue. (**C** to **E**) MS-MS spectra comparing experimental data (red) to the reference spectra (blue) used to identify polyamine metabolites. Metabolite structures are shown at right along with fold enrichment. (**F**) Extracted ion chromatograms showing elution peaks of polyamine analytical standards and polyamines in microbe-conditioned media. *m*/*z* ratios are for the dansylated polyamines. Peaks are normalized to their maxima. (**G**) Enrichment of polyamines in *E. coli*– and *E. faecalis*–conditioned media compared to unconditioned media. Detected levels of each metabolite in microbe conditioned media were divided by the mean level of the metabolite detected in nine replicates of unconditioned media. Error bars represent mean ± SD. Each point represents one replicate, with three technical replicates performed across each of three biological replicates.

Having identified neurons that function in a chemosensory circuit that guides *C. elegans* to nutritive *E. coli*, we next sought to determine the identities of molecular cues sensed by this circuit. We used untargeted liquid chromatography–mass spectrometry (LC-MS) to profile the molecular composition of *E. coli*– and *E. faecalis*–conditioned media (*43*). Principal components analysis showed that media conditioned by nutritive *E. coli* and pathogenic *E. faecalis* could be distinguished from each other and from unconditioned media on the basis of their measured metabolomes (Fig. 3A). A comparison of *E. coli*- and *E. faecalis*-conditioned media identified 282 metabolites that were at least two-fold enriched in *E. coli*-conditioned media (Fig. 3B and table S1). Among the most enriched metabolites was an acetylated form of the polyamine cadaverine, which displayed a log_2_ fold enrichment over 7 (Fig. 3C). Upon examination of those metabolites most enriched and with the highest levels of significance, *N*-acetylcadaverine was surpassed only by a group of di- and tripeptides (fig. S6). Inspection of the list of *E. coli*–enriched metabolites revealed high enrichment of two other acetylated polyamines, *N*-acetylputrescine and N8-acetylspermidine (Fig. 3, B to E).

The assay used for untargeted metabolomics does not readily detect nonacetylated polyamines due to the poor ionization of these compounds. To determine whether *E. coli*–conditioned medium was enriched for nonacetylated polyamines as well, we derivatized metabolites in microbe-conditioned media by dansylation, which adds a large ionizable moiety to amine-containing compounds (Fig. 3F) (*44*, *45*). Using this approach, we were able to perform targeted metabolomics on *E. coli*– and *E. faecalis*–conditioned media for dansylated polyamines and dansylated acetyl-polyamines. Compared to unconditioned media, *E. coli*–conditioned medium is highly enriched for both acetylated and nonacetylated polyamines, while *E. faecalis*–conditioned medium is not enriched for polyamines (Fig. 3G).

**Fig. 4.**
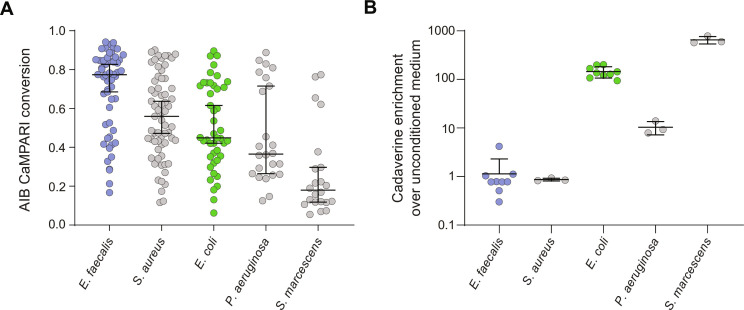
AIB inhibition and polyamine enrichment are correlated in diverse microbes. (**A**) CaMPARI signals in animals exposed to *E. faecalis* (*n* = 54 cells, data are replotted from Fig. 2C), *S. aureus* (*n* = 65 cells), *E. coli* (*n* = 44 cells), *P. aeruginosa* (*n* = 24 cells), or *Serratia marcescens* (*n* = 22 cells). Error bars represent median with 95% confidence interval. Each point represents one cell. (**B**) Enrichment of cadaverine in microbe conditioned media compared to unconditioned media. Detected levels of cadaverine in each microbe conditioned medium were divided by the mean level of the metabolite detected in nine replicates of unconditioned media. *E. faecalis* and *E. coli* data are replotted from Fig. 3G. Error bars represent mean ± SD. Each point represents one replicate.

We tested whether there was a correlation between how microbes activate the *C. elegans* microbe-sensing circuit and their polyamine content. We assessed a panel of microbial species that elicit different behavioral responses from *C. elegans* and that are differently pathogenic (*9*, *13*, *46*) for their effects on AIB activity. Exposure to *Serratia marcescens* and *Pseudomonas aeruginosa* caused marked inhibition of AIBs as reported by CaMPARI (Fig. 4A). These bacterial species are, such as *E. coli*, attractive to naïve animals. Exposure to another species, *Staphylococcus aureus*, did not inhibit AIB neurons (Fig. 4A), and this species, such as *E. faecalis*, is not attractive to *C. elegans* (*13*). We used targeted LC-MS analysis of microbial polyamine synthesis to measure production of cadaverine, which was polyamine species most enriched in *E. coli* compared to *E. faecalis* (Fig. 3G). This revealed that cadaverine production varied between the microbial species tested and was produced at higher levels by microbes that are innately attractive to naïve *C. elegans* (Fig. 4B). These data suggest that polyamines such as cadaverine might be sensed by *C. elegans* to control microbe response behaviors.

**Fig. 5.**
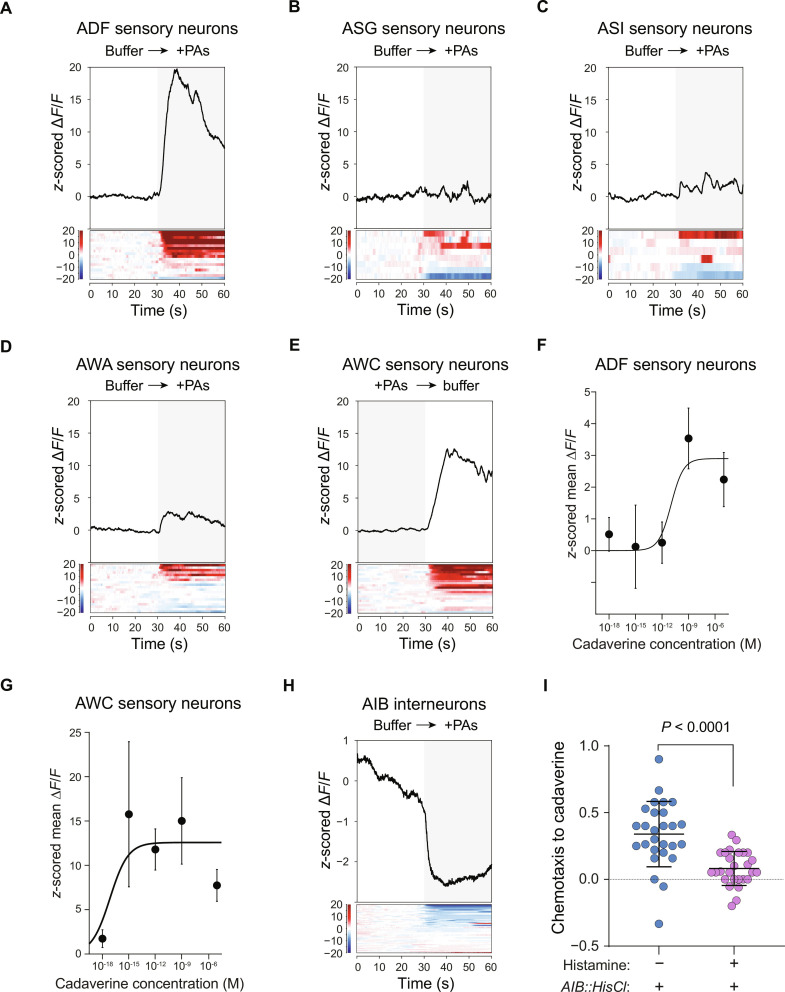
Polyamine metabolites are sensed by a subset of the neurons tuned to detect *E. coli* and suffice to instruct *C. elegans* chemotaxis. (**A** to **E**) Responses of *E. coli*–responsive neurons to a cocktail of polyamines comprising equimolar (25 mM) amounts of cadaverine, putrescine, and spermidine plus 2.5 mM spermine. Data from 18 trials of ADFs, 8 trials of ASGs, 6 trials of ASIs, 20 trials of AWAs, and 22 trials of AWCs are plotted. (**F**) Dose-response curve of responses of ADF neurons to cadaverine. Points indicate the mean, and error bars represent SEM. Data from 28 trials of 10^−5^ M, 26 trials of 10^−9^ M, 10 trials of 10^−12^ M, 6 trials of 10^−15^ M, and 8 trials of 10^−18^ M are plotted. (**G**) Dose-response curve of responses of AWC neurons to cadaverine. Points indicate the mean, and error bars represent SEM. Data from 20 trials of 10^−5^ M, 20 trials of 10^−9^ M, 14 trials of 10^−12^ M, 14 trials of 10^−15^ M, and 26 trials of 10^−18^ M are plotted. (**H**) Responses of AIB interneurons to the cocktail of polyamine odorants used in (A). Data from 50 trials are plotted. (**I**) Chemotaxis indices computed from behavioral responses to cadaverine of transgenic animals expressing HisCl in AIB interneurons either with or without histamine. In the presence of histamine, AIB interneurons are silenced. Data are plotted from 27 trials of each condition. Each trial used 20 animals. Student’s *t* test was used to compute the indicated *P* value. Error bars represent mean ± SD. Each point represents one trial. In (A) to (G), traces of the mean *z*-scored signals are shown above heatmaps, showing the *z*-scored signals from individual trials. PAs, polyamines.

To determine whether polyamine metabolites are sensed by *C. elegans* as indicators of nutritive microbes, we measured activity of chemosensory neurons as we presented a blend of the major *E. coli*–enriched polyamines cadaverine, putrescine, spermidine, and a minor polyamine, spermine. Of the five neuron types that preferentially responded to *E. coli*–conditioned media, two responded to polyamines (Fig. 5, A to E). ADF neurons responded to presentation of polyamines (Fig. 5A), and AWC neurons responded to removal of polyamine stimuli (Fig. 5E), mirroring their responses to *E. coli*–conditioned media. Because cadaverine was the most highly enriched polyamine in *E. coli*–conditioned media, we tested whether it was sufficient to activate ADF and AWC neurons. Cadaverine on its own sufficed to activate ADF neurons (fig. S7). We found that AWC neurons were remarkably sensitive to cadaverine and displayed responses to 1-fM cadaverine that were as large as the responses we observed to the polyamine blend, which contained cadaverine at millimolar concentrations. In contrast, sensitivity of ADF neurons to cadaverine decreased after 1 nM (Fig. 5, F and G, and fig. S8). We concluded that polyamine metabolites, most notably cadaverine, were sensed by two of the five classes of sensory neuron that are associated with detection of nutritive microbes.

We next tested whether the subset of chemosensory neurons that responded to polyamine metabolites were able to elicit responses in AIB interneurons, which are essential for efficient chemotaxis to nutritive microbes (Fig. 2I). The polyamine blend that was sensed by ADF and AWC neurons caused rapid inhibition of AIB interneurons, indicating that olfactory responses to polyamine metabolites sufficed to regulate these key interneurons in the microbe-sensing circuit (Fig. 5H). Cadaverine was revealed to be the major differentially expressed polyamine by the targeted metabolomics (Fig. 4G) and was also sufficient to activate ADF and AWC sensory neurons (Fig. 5, A and E). A chemotaxis assay revealed that cadaverine is also perceived by *C. elegans* as inherently attractive (Fig. 5I). Attraction of *C. elegans* to *E. coli*–associated cadaverine was abolished by chemogenetic silencing of AIBs (Fig. 5I). To identify the contribution of polyamines to microbial chemotaxis, we supplemented *E. faecalis* conditioned media with the polyamine mixture and found that chemotaxis to this blend was significantly improved over chemotaxis to *E. faecalis* alone (fig. S9).

**Fig. 6.**
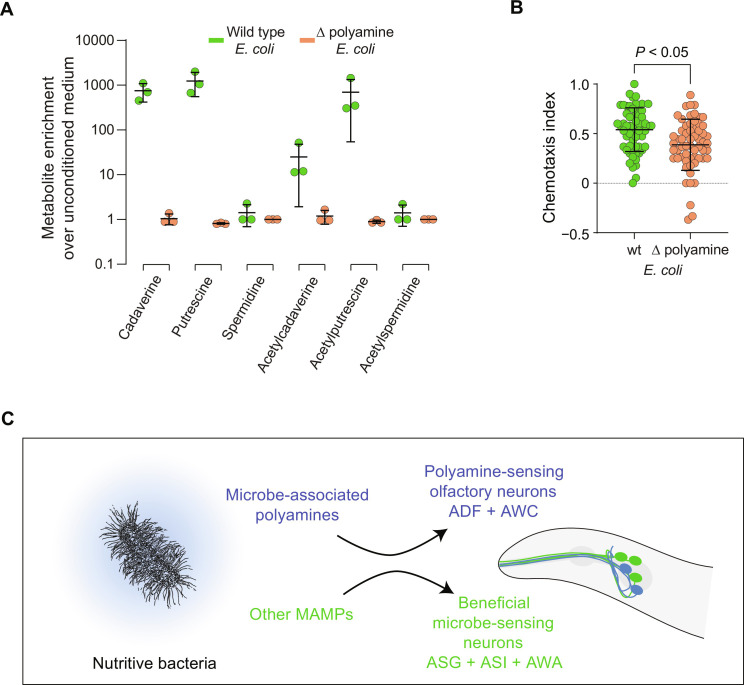
Mutating the polyamine synthesis pathway reduces attraction to nutritive bacteria. (**A**) Enrichment of polyamines in wild-type and polyamine-deficient (mutant) *E. coli*–conditioned media compared to unconditioned media. Detected levels of each metabolite in microbe-conditioned media were divided by the mean level of the metabolite detected in three replicates of unconditioned media. Error bars represent mean ± SD. Each point represents one replicate. wt, wild type; mut, mutant. (**B**) Chemotaxis indices computed from behavioral responses to wild-type or polyamine-deficient (mutant) *E. coli*–conditioned media (*n* = 60 trials for each bacterium). Student’s *t* test was used to compute the indicated *P* value. Error bars represent mean ± SD. Each point represents one trial. (**C**) Model of chemosensory detection of nutritive bacteria using chemosensation of polyamines and chemosensation of other microbe-specific metabolites. MAMPs, microbe-associated molecular patterns.

To what extent do polyamines determine the attraction of *C. elegans* to *E. coli*? To answer this question, we used a strain of *E. coli* carrying mutations in genes required for polyamine synthesis (*47*). We used targeted measurements of endogenous polyamines to confirm that this mutant strain was devoid of polyamines when grown in minimal media (Fig. 6A). We then compared chemotaxis of *C. elegans* to media conditioned by wild-type or polyamine-deficient *E. coli*. Attraction of *C. elegans* to media conditioned by polyamine-deficient *E. coli* was significantly reduced in comparison to attraction to media conditioned by wild-type *E. coli* (Fig. 6B). Polyamine-deficient *E. coli* were still able to synthesize some attractive cues as indicated by a chemotaxis index that was, on average, positive (Fig. 6B). These data further show that endogenous polyamines are a significant component of the metabolite blend sensed by *C. elegans* to identify and navigate to nutritive *E. coli*. We suggest that our study supports a model in which *C. elegans* evaluates the polyamine content of the secreted microbial metabolome to make rapid decisions about microbe quality and instruct foraging behavior (Fig. 6C).

## DISCUSSION

Polyamines are well known as ethologically relevant odorants that potently elicit innate animal behaviors. Exposure of mice to cadaverine, putrescine, and spermidine elicits hallmarks of anxiety and avoidance behaviors (*48*). Similarly, zebrafish sense cadaverine and putrescine as highly aversive odorants (*49*). Other animals are, such as *C. elegans*, attracted to these polyamines. Cadaverine and putrescine are sensed as appetitive cues by rats, goldfish, and *Drosophila melanogaster* (*50*–*52*). It is evident that the olfactory mechanisms that detect polyamines to mediate these different innate behaviors in these highly diverged species have independently emerged multiple times during animal evolution. Odorant receptors directly activated by polyamines in the insect olfactory system are polyamine-gated ion channels distantly related to ionotropic neurotransmitter receptors in the nervous system (*50*). Vertebrates sense polyamines using trace amine-associated receptors (TAARs), which are a vertebrate-specific clade of G protein–coupled receptors descended from metabotropic catecholamine and monoamine receptors (*53*). The *C. elegans* genome lacks homologs of either TAARs or insect odorant receptors, indicating that nematodes have acquired their own mechanisms for olfactory detection of polyamines.

Our study indicates that *C. elegans* has at least two distinct mechanisms for polyamine sensing. The chemosensory neurons that respond to polyamine odorants—ADF and AWC—use different signaling pathways for sensory transduction (*21*, *22*, *54*), indicating that they use distinct odorant receptors. Our study found that ADF and AWC neurons are also differently sensitive to the polyamine cadaverine, with AWCs displaying exceptionally high sensitivity to this polyamine. Could the receptors used by ADF and AWC neurons to sense polyamines be conserved between nematodes and vertebrates? The recent discovery that the vertebrate histamine receptor HRH4 is a non-olfactory receptor for polyamines produced by gut microbes (*55*) illustrates that much remains to be known about how polyamine microbial metabolites are sensed by animal hosts. Polyamines are produced by the vertebrate microbiome and modulate the intestinal mucosa and resident macrophages through unknown mechanisms (*56*). An intriguing possibility is that vertebrate homologs of nematode polyamine receptors function in detecting microbes in internal milieus. Further study is required to identify the receptors used by *C. elegans* to sense polyamines and determine if they are conserved in other animals.

Polyamine sensing is only part of what is likely a sophisticated sensory mechanism *C. elegans* uses to identify microbes before ingesting them. However, *C. elegans* was still attracted to mutant *E. coli* bacteria that lack detectable polyamines (Fig. 6B), indicating that *C. elegans* senses other metabolites, perhaps including the highly enriched di- and tripeptides (fig. S6) to identify and respond to these microbes. This study identified eight types of chemosensory neuron that are engaged by microbe-specific cues and demonstrated that microbe identity is encoded by activity of subsets of these eight neurons. A simple binary encoding scheme using these eight neuron types would generate 2^8^ or 256 different codes and thereby permit innate recognition of hundreds of microbial species. Given that *C. elegans* has 16 classes of chemosensory neuron and that these neurons are not limited to binary “on” or “off” states, the number of microbe identities that could be encoded is many orders of magnitude higher. Our data suggest that the AIB interneurons that integrate this sensory input may evaluate the attractiveness of a stimulus and instruct appropriate behaviors based on the intensity of sensory input received (*42*). This supports the encoding of information beyond binary on or off states in this olfactory circuit. It should be noted that, to date, the extent to which *C. elegans* can discriminate between olfactory stimuli has not been tested experimentally. The integration of information from sensory neurons by different downstream circuits could permit innate behavioral responses to specific microbes to be defined by organized assembly of chemosensory and motor circuits.

The prevalence and diversity of mechanisms for olfactory detection of polyamines in the animal kingdom suggests an important role for this chemosensory modality. Polyamine odorants are associated with the actions of microbes on decaying biomass as indicated by their names. This study and others (*57*) indicates that different microbial species produce different complements of polyamine metabolites. Our study shows how polyamine metabolites are recognized by *C. elegans* as an indicator of nutritive microbes, and we suggest that polyamine sensing might be widely used by animals to innately recognize microbes and allow animals to discriminate between those that are pathogenic and those that are beneficial.

## MATERIALS AND METHODS

### Nematode culture, strains, and maintenance

*C. elegans* hermaphrodites were maintained at 20°C on nematode growth medium (NGM) agar supplemented with a thin layer of *E. coli* strain OP50 using standard methods (*58*). The wild-type strain used in this study was Bristol N2. A complete list of strains used in this study is presented in table S3.

### Genotyping mutant *C. elegans*

The *lite-1(ce314)* allele was detected by PCR primers that selectively amplified wild-type or *ce314* sequences. The *tax-4(p678)* and *osm-9(ky10)* alleles were detected similarly. Primers for allele-specific PCR were designed using SNAPER (*59*). A list of primers used in this study is presented in table S4.

### Generation of transgenes and plasmids

All plasmids were generated using Gibson cloning (*60*). A full list of plasmids constructed for this study is presented in table S5.

### Generation of transgenic *C. elegans* strains

Transgenic *C. elegans* strains were generated by microinjection (*61*). Some transgenes were integrated by x-ray irradiation (50 grays). Integrants were outcrossed one to three times with the N2 wild type. Table S3 lists transgenics generated by this study.

### Measuring neural activity with CaMPARI

Animals were corralled in plexiglass rings whose bottoms were painted with glycerol to form a high-osmolarity barrier. The internal diameter of corrals was 2 cm. CaMPARI conversion was performed with a 405-nm light-emitting diode (LED) (Thorlabs M405L3) that was collimated to generate a 2.6-cm diameter beam centered on corrals. For each trial, 10 to 20 young adult animals were picked into the center of the corral and irradiated for the duration of the experiment. The intensity of light used for photoconversion was 0.26 mW per mm^2^. Animals were exposed to conversion light for 1 min and then transferred to a slide, paralyzed in levamisole, and imaged on a line-scanning confocal microscope (Zeiss LSM700). The microscope used to image photoconverted CaMPARI was calibrated by determining excitation light power and detector-gain settings that reported increases in green fluorescence emissions equal to increases in red fluorescence emissions within CaMPARI animals that were imaged before and after photoconversion [as described in (*35*)].

### Fluorescence microscopy

Adult animals were anesthetized with 30 mM sodium azide and mounted on 2% agarose pads made with M9 buffer (22 mM KH_2_PO_4_, 22 mM Na_2_HPO_4_, 85 mM NaCl, and 1 mM MgSO_4_). Fluorescence and differential interference contrast micrographs were obtained using an LSM700 laser-scanning confocal microscope (Zeiss, Oberkochen, Germany) with a 40× objective (1.4 numerical aperture). Maximum projection images of image stacks were generated with the Fiji ImageJ distribution (*62*).

### Statistical analysis

Statistical tests were performed with Prism (Graphpad Software; San Diego, CA). Kolmogorov-Smirnov tests were used for CaMPARI data, and either Student’s *t* test or analysis of variance (ANOVA) was used for behavior data.

### Calcium imaging

Animals were immobilized in a microfluidics device (*24*) without paralytic. They were imaged on a spinning disk confocal microscope (Nikon) at 10 frames/s. A single focal plane was imaged for each trial to reduce merging of fluorescence from multiple planes. For some trials, fluorescein was added to one stimulus reservoir, and rhodamine was added to the other to monitor stimulus transitions. Once it was clear that transitions were accurately recorded, the protocol was updated such that only rhodamine was used in one of the stimulus reservoirs. Trials consisted of alternating blocks of stimulus presentation, each 1 min long. A signal generator (Rigol) was used to control the valve that defined stimulus presentation. Trial length was either 3 min in total using the structure A-B-A or 5 min using A-B-A-B-A.

Initial analysis and extraction of traces was performed in ImageJ. Stimulus fluorescence information was extracted from each recording to identify transition points. Recordings were then cropped and registered using the StackReg plugin of ImageJ to reduce movement. Regions of interest (ROIs) were drawn by hand around cell bodies, including a background ROI drawn inside the animal’s head that did not overlap with any fluorescent structure. ROIs were drawn to avoid overlap between cells. Trials were excluded if registration could not reduce movement sufficiently to remove ROI overlap or if recorded stimuli did not closely match the protocol. Fluorescence data were measured from all cellular and background ROIs and imported into MATLAB. Stimulus transitions were identified and 1-min windows surrounding those transitions were identified for every trial, with 30 s preceding each transition and 30 s following it. Each 60-s window was *z*-scored with respect to the first 30 s. These *z*-scored trials were then averaged across the transition type (for example, A-B or B-A). Trials were *z*-scored to control for stimulus-independent fluctuations in neural activity. Non–*z*-scored ∆*F*/*F* data are plotted in figs. S10 and S11.

### Occupancy behavior assay

NGM plates (6 cm) were seeded with 20 μl of bacterial culture that had been grown at 37°C with shaking for 8 hours. Seeded plates were allowed to grow for 16 hours, so that a thin lawn of bacteria would develop. Thirty synchronized adult animals were picked onto a transfer plate to remove adherent bacteria and then transferred to the assay plate midway between the edge of the lawn and the edge of the plate. The plate was then placed in a tracking rig, which illuminated it with a custom-build red LED ring light, as described in (*35*), and recorded video at 1 f/s for 10 min using a one-megapixel charge-coupled device camera (UniBrain, San Ramon, CA).

Videos were analyzed by identifying every frame at which an entry or an exit event occurred. Entry time was defined as when the nose of an animal made contact with the bacterial lawn if the animal then proceeded into the lawn, such that at least 75% of its body was in the lawn. Exit times were defined as the last frame at which the tail of the animal was in contact with the bacterial lawn. Instantaneous occupancy was calculated as frame-to-frame differences between entry events and occupancy events but could not always be validated by ground truth due to the low contrast between animals and bacteria at the edges of the bacterial lawns. End-point occupancy data were acquired by removing the plates from the tracker after the 10 min had elapsed and counting the number of animals present in the bacterial lawn using a dissecting microscope.

### Assaying chemotaxis behavior

Bacteria were cultured as above for the occupancy behavior assay. Four spots equidistant from the center were marked on each 6-cm NGM plate, and two spots opposite from each other were selected for seeding. Each spot was seeded with 20 μl of bacterial culture and allowed to grow with the lid ajar for 16 hours. On the basis of these four spots, each plate was divided into quadrants. Five min before the assay, 1 μl of 100 mM levamisole with green food coloring added for visualization was dropped onto each of the four spots. Once it was clear that the levamisole paralytic was not spreading beyond the intended area, the green food coloring was excluded. Twenty synchronized adult animals were picked onto a transfer plate to reduce transfer of bacteria, as above, and then moved to the center of the assay plate. After 10 min passed, the number of animals in each quadrant was counted, excluding any animals that had not moved from the center of the plate. A chemotaxis index was calculated as follows: (# of animals in lawn quadrants − # of animals in control quadrants)/(# of animals in lawn quadrants + # of animals in control quadrants).

Chemotaxis to conditioned media or polyamine solutions was assayed on chemotaxis medium plates using the above method. Conditioned media were diluted 1:10 in S-basal solution, and 20 μl of conditioned media were spotted as above, 16 hours before the trial. For comparison of *E. coli* to *E. faecalis*, *E. coli* strain OP50 and *E. faecalis* strain OG1RF were used. To determine the effect of polyamines on chemotaxis, *E. coli* strain HT779 was used as the wild type and *E. coli* strain HT873 was used as the polyamine-deficient strain. For experiments in which a mixture of polyamines was used, the mixture comprised 12.5 mM spermidine (Sigma-Aldrich), 12.5 mM cadaverine (Sigma-Aldrich), 12.5 mM putrescine dihydrochloride (Sigma-Aldrich), and 1.25 mM spermine (Sigma-Aldrich). When polyamines were added to conditioned media, 5 μl of polyamine solution was added immediately following the 20 μl of conditioned media. When the polyamine mixture was assessed alone, 20 μl of S-basal was spotted in place of conditioned media before addition of 5 μl of polyamine solution to control for moisture content. For experiments in which a cadaverine solution was used, the solution was 2.5 mM cadaverine. A 20 μl of cadaverine solution was spotted as above, 16 hours before the trial. Under these conditions, a spot of 12.5 mM odorants generates a gradient that approximately ranges from 1 μM at the center of the plate to 12.5 μM at the spot, according to a typical diffusion coefficient for molecules of similar sizes to polyamines and previous reports (*40*).

### Assaying lawn retention behavior

A template was designed, such that a 6-cm plate would have a spot 1 cm in diameter at the center and then a ring of six spots spaced at the vertices of a regular hexagon such that the vertices are each 1.75 cm from the center of the plate. A 20 μl of OP50 *E. coli* was spotted at each of these six peripheral spots for every trial. The center was spotted with 20 μl of either *E. coli* or OG1RF *E. faecalis* depending on the trial. As with the chemotaxis assay, 20 synchronized adult animals were picked onto a transfer plate to remove adherent bacteria and then transferred to the central lawn. After 10 min, the number of animals remaining in the central lawn was counted.

### Chemogenetic silencing

Histamine-containing plates were prepared by adding 10 ml of 1 M histamine dihydrochloride to 1 liter of NGM media while still liquid. The NGM medium was prepared in a batch of 2 liters so that paired plates could be poured. Both chemotaxis and lawn retention assays were performed as described above, running trials on histamine and paired NGM plates as paired controls. The procedure for chemogenetic silencing during chemotaxis to cadaverine was identical to above, except that chemotaxis medium was prepared instead of NGM.

### Optogenetic silencing

The movement of transgenics expressing Archaerhodopsin in AIBs was recorded at 3 f/s while they experienced randomly timed 1-s pulses of green light, such that at every timestep, the probability of initiating a light pulse was 0.1. Trajectories from 30 animals were collected, with animals switched out every 15 min. Uniform datasets were extracted from the complete dataset and were separated into ON and OFF light samples as follows. ON samples had a 1-s light pulse at the onset of the trajectory; OFF samples did not. Further, OFF samples were restricted such that there were no pulses in the entire 20-s trajectory. ON and OFF paths were centered to start at the origin and reoriented, so that the initial measured body angle at the first timestep was 0° relative to the positive *x* axis. Then, total distance traveled along the *x* axis was calculated for each ON and OFF sample to produce the plot in Fig. 2K. The total distance traveled was calculated for fig. S5.

### Metabolomics

Samples were extracted and subjected to LC-MS analysis based on a previously described method (*43*). Conditioned medium was extracted to a measured aliquot of 200 μl media/ml of LC-MS grade methanol. Samples were then homogenized using a BeadBlaster (Benchmark Scientific) and centrifuged (21,000*g* for 3 min at 4°C). Supernatant (1800 μl) was dried down via speed vacuum concentrations and reconstituted in 200 μl of LC-MS grade water. The LC column was a Millipore ZIC-pHILIC (2.1 mm by 150 mm, 5 μm) coupled to a Dionex Ultimate 3000 system, and the column oven temperature was set to 25°C for the gradient elution. A flow rate of 100 μl/min was used with the following buffers: (A) 10 mM ammonium carbonate in water (pH 9.0) and (B) neat acetonitrile. The gradient profile was as follows: 80 to 20% B (0 to 30 min), 20 to 80% B (30 to 31 min), 80 to 80% B (31 to 42 min). Injection volume was set to 1 μl for all analyses (42 min total run time per injection). MS analyses were carried out by coupling the LC system to a Thermo Q Exactive HF mass spectrometer operating in heated electrospray ionization mode. Spray voltage for both positive and negative modes was 3.5 kV, and capillary temperature was set to 320°C with a sheath gas rate of 35, auxiliary gas of 10, and max spray current of 100 μA. The full MS scan for both polarities used 120,000 resolution with an automatic gain control (AGC) target of 3 × 10^6^ and a maximum injection time (IT) of 100 ms, and the scan range was from 67 to 1000 mass/charge ratio (*m*/*z*). Tandem MS spectra for both positive and negative mode used a resolution of 15,000, AGC target of 1 × 10^5^, maximum IT of 50 ms, isolation window of 0.4 *m*/*z*, isolation offset of 0.1 *m*/*z*, fixed first mass of 50 *m*/*z*, and three-way multiplexed normalized collision energies of 10, 35, and 80. The resulting Thermo RAW files were converted to mzXML format using ReAdW.exe version 4.3.1 to enable peak detection and quantification. The centroided data were searched using an in-house python script Mighty_skeleton version 0.0.2, and peak heights were extracted from the mzXML files. Annotations were performed using NIST14MS/MS (*63*) and METLIN (2017) (*64*) tandem mass spectral libraries. Metabolite peaks were extracted on the basis of the theoretical *m*/*z* of the expected ion type, e.g., [M + H]+, with a ±5 parts per million tolerance, and a ±7.5-s peak apex retention time tolerance within an initial retention time search window of ±0.5 min across the study samples. The resulting data matrix of metabolite intensities for all samples and blank controls was processed with an in-house statistical pipeline Metabolyze version 1.0, and final peak detection was calculated on the basis of a signal-to-noise ratio of 3× compared to blank controls, with a floor of 10,000 (arbitrary units).

For the targeted LC/MS method, samples were dansylated according to the following protocol, adapted from (*44*, *45*). A 300 μl of a dansyl-chloride solution [5 mg of dansyl-chloride (Sigma-Aldrich) per 1 ml of acetone (Thermo Fisher Scientific)] was added to 100 μl of conditioned medium. A 100 μl of saturated sodium carbonate solution was added, and the mixture was vortexed briefly. Samples were incubated in the dark for 18 hours. Following incubation, 50 μl of proline solution (100 mg/ml) was added to each sample. Solutions (1 M) of acetylcadaverine (Cayman), acetylputrescine (Sigma-Aldrich), acetylspermidine (Sigma-Aldrich), cadaverine (Sigma-Aldrich), putrescine (Sigma-Aldrich), and spermidine (Sigma-Aldrich) were dansylated and analyzed using the protocol described above for use as standards. The *m*/*z* ratios and elution spectra of the dansylated standards were identified and used to target the analysis of samples. Conditioned media samples were then analyzed using the protocol described above.

### Metabolite enrichment analysis

Targeted LC-MS intensity values for each polyamine metabolite were used. For comparisons to OP50 *E. coli*, OG1RF *E. faecalis*, NCTC8325 *S. aureus*, PA14 *P. aeruginosa*, and DB10 *S. marcescens*, the unconditioned medium was Luria Broth. Each individual microbial replicate was divided by the mean of all unconditioned medium replicates to obtain the fold enrichment. For comparisons to HT779 *E. coli* and HT873 *E. coli*, the unconditioned medium was M9 minimal medium. Any values measured below the detection threshold were replaced with the detection threshold.

## Supplementary Materials

### This PDF file includes:

Figs. S1 to S11 Legends for tables S1 and S2 Tables S3 to S5 Legends for data files S1 to S7

## References

[1] G. den Besten, K. Lange, R. Havinga, T. H. van Dijk, A. Gerding, K. van Eunen, M. Muller, A. K. Groen, G. J. Hooiveld, B. M. Bakker, D. J. Reijngoud, Gut-derived short-chain fatty acids are vividly assimilated into host carbohydrates and lipids. Am. J. Physiol. Gastrointest. Liver Physiol. 305, G900–G910 (2013). 24136789 10.1152/ajpgi.00265.2013

[2] J. M. Natividad, A. Agus, J. Planchais, B. Lamas, A. C. Jarry, R. Martin, M. L. Michel, C. Chong-Nguyen, R. Roussel, M. Straube, S. Jegou, C. McQuitty, M. Le Gall, G. da Costa, E. Lecornet, C. Michaudel, M. Modoux, J. Glodt, C. Bridonneau, B. Sovran, L. Dupraz, A. Bado, M. L. Richard, P. Langella, B. Hansel, J. M. Launay, R. J. Xavier, H. Duboc, H. Sokol, Impaired aryl hydrocarbon receptor ligand production by the gut microbiota is a key factor in metabolic syndrome. Cell Metab. 28, 737–749.e4 (2018). 30057068 10.1016/j.cmet.2018.07.001

[3] P. Strandwitz, K. H. Kim, D. Terekhova, J. K. Liu, A. Sharma, J. Levering, D. McDonald, D. Dietrich, T. R. Ramadhar, A. Lekbua, N. Mroue, C. Liston, E. J. Stewart, M. J. Dubin, K. Zengler, R. Knight, J. A. Gilbert, J. Clardy, K. Lewis, GABA-modulating bacteria of the human gut microbiota. Nat. Microbiol. 4, 396–403 (2019). 30531975 10.1038/s41564-018-0307-3 PMC6384127

[4] A. Zečić, I. Dhondt, B. P. Braeckman, The nutritional requirements of *Caenorhabditis elegans*. Genes Nutr. 14, 15 (2019). 31080524 10.1186/s12263-019-0637-7 PMC6501307

[5] P. Dirksen, A. Assié, J. Zimmermann, F. Zhang, A. M. Tietje, S. A. Marsh, M. A. Félix, M. Shapira, C. Kaleta, H. Schulenburg, B. S. Samuel, CeMbio - The *Caenorhabditis elegans* microbiome resource. G3 10, 3025–3039 (2020). 32669368 10.1534/g3.120.401309 PMC7466993

[6] B. S. Samuel, H. Rowedder, C. Braendle, M. A. Félix, G. Ruvkun, *Caenorhabditis elegans* responses to bacteria from its natural habitats. Proc. Natl. Acad. Sci. U.S.A. 113, E3941–E3949 (2016). 27317746 10.1073/pnas.1607183113 PMC4941482

[7] H. Schulenburg, M. A. Félix, The natural biotic environment of *Caenorhabditis elegans*. Genetics 206, 55–86 (2017). 28476862 10.1534/genetics.116.195511 PMC5419493

[8] C. L. Kurz, J. J. Ewbank, Caenorhabditis elegans for the study of host-pathogen interactions. Trends Microbiol. 8, 142–144 (2000). 10707068 10.1016/s0966-842x(99)01691-1

[9] Y. Zhang, H. Lu, C. I. Bargmann, Pathogenic bacteria induce aversive olfactory learning in *Caenorhabditis elegans*. Nature 438, 179–184 (2005). 16281027 10.1038/nature04216

[10] X. Jin, N. Pokala, C. I. Bargmann, Distinct circuits for the formation and retrieval of an imprinted olfactory memory. Cell 164, 632–643 (2016). 26871629 10.1016/j.cell.2016.01.007 PMC5065712

[11] J. D. Meisel, D. H. Kim, Behavioral avoidance of pathogenic bacteria by *Caenorhabditis elegans*. Trends Immunol. 35, 465–470 (2014). 25240986 10.1016/j.it.2014.08.008

[12] R. S. Moore, R. Kaletsky, C. T. Murphy, Piwi/PRG-1 argonaute and TGF-β mediate transgenerational learned pathogenic avoidance. Cell 177, 1827–1841.e12 (2019). 31178117 10.1016/j.cell.2019.05.024 PMC7518193

[13] J. Singh, A. Aballay, Microbial colonization activates an immune fight-and-flight response via neuroendocrine signaling. Dev. Cell 49, 89–99.e4 (2019). 30827896 10.1016/j.devcel.2019.02.001 PMC6456415

[14] B. B. Shtonda, L. Avery, Dietary choice behavior in *Caenorhabditis elegans*. J. Exp. Biol. 209, 89–102 (2006). 16354781 10.1242/jeb.01955 PMC1352325

[15] A. Lin, S. Qin, H. Casademunt, M. Wu, W. Hung, G. Cain, N. Z. Tan, R. Valenzuela, L. Lesanpezeshki, V. Venkatachalam, C. Pehlevan, M. Zhen, A. D. T. Samuel, Functional imaging and quantification of multineuronal olfactory responses in *C. elegans*. Sci. Adv. 9, eade1249 (2023). 36857454 10.1126/sciadv.ade1249 PMC9977185

[16] A. Zaslaver, I. Liani, O. Shtangel, S. Ginzburg, L. Yee, P. W. Sternberg, Hierarchical sparse coding in the sensory system of *Caenorhabditis elegans*. Proc. Natl. Acad. Sci. U.S.A. 112, 1185–1189 (2015). 25583501 10.1073/pnas.1423656112 PMC4313814

[17] J. D. Meisel, O. Panda, P. Mahanti, F. C. Schroeder, D. H. Kim, Chemosensation of bacterial secondary metabolites modulates neuroendocrine signaling and behavior of *C. elegans*. Cell 159, 267–280 (2014). 25303524 10.1016/j.cell.2014.09.011 PMC4194030

[18] E. Pradel, Y. Zhang, N. Pujol, T. Matsuyama, C. I. Bargmann, J. J. Ewbank, Detection and avoidance of a natural product from the pathogenic bacterium *Serratia marcescens* by *Caenorhabditis elegans*. Proc. Natl. Acad. Sci. U.S.A. 104, 2295–2300 (2007). 17267603 10.1073/pnas.0610281104 PMC1892944

[19] S. E. Worthy, G. L. Rojas, C. J. Taylor, E. E. Glater, Identification of Odor Blend Used by *Caenorhabditis elegans* for Pathogen Recognition. Chem. Senses 43, 169–180 (2018). 29373666 10.1093/chemse/bjy001 PMC6018680

[20] A. Tran, A. Tang, C. T. O'Loughlin, A. Balistreri, E. Chang, D. Coto Villa, J. Li, A. Varshney, V. Jimenez, J. Pyle, B. Tsujimoto, C. Wellbrook, C. Vargas, A. Duong, N. Ali, S. Y. Matthews, S. Levinson, S. Woldemariam, S. Khuri, M. Bremer, D. K. Eggers, N. L'Etoile, L. C. Miller Conrad, M. K. VanHoven, *C. elegans* avoids toxin-producing Streptomyces using a seven transmembrane domain chemosensory receptor. eLife 6, e23770 (2017). 28873053 10.7554/eLife.23770 PMC5584987

[21] H. A. Colbert, T. L. Smith, C. I. Bargmann, OSM-9, a novel protein with structural similarity to channels, is required for olfaction, mechanosensation, and olfactory adaptation in Caenorhabditis elegans. J. Neurosci. 17, 8259–8269 (1997). 9334401 10.1523/JNEUROSCI.17-21-08259.1997 PMC6573730

[22] H. Komatsu, I. Mori, J. S. Rhee, N. Akaike, Y. Ohshima, Mutations in a cyclic nucleotide-gated channel lead to abnormal thermosensation and chemosensation in C. elegans. Neuron 17, 707–718 (1996). 8893027 10.1016/s0896-6273(00)80202-0

[23] T. W. Chen, T. J. Wardill, Y. Sun, S. R. Pulver, S. L. Renninger, A. Baohan, E. R. Schreiter, R. A. Kerr, M. B. Orger, V. Jayaraman, L. L. Looger, K. Svoboda, D. S. Kim, Ultrasensitive fluorescent proteins for imaging neuronal activity. Nature 499, 295–300 (2013). 23868258 10.1038/nature12354 PMC3777791

[24] N. Chronis, M. Zimmer, C. I. Bargmann, Microfluidics for in vivo imaging of neuronal and behavioral activity in *Caenorhabditis elegans*. Nat. Methods 4, 727–731 (2007). 17704783 10.1038/nmeth1075

[25] S. H. Chalasani, N. Chronis, M. Tsunozaki, J. M. Gray, D. Ramot, M. B. Goodman, C. I. Bargmann, Dissecting a circuit for olfactory behaviour in *Caenorhabditis elegans*. Nature 450, 63–70 (2007). 17972877 10.1038/nature06292

[26] J. G. White, E. Southgate, J. N. Thomson, S. Brenner, The structure of the nervous system of the nematode *Caenorhabditis elegans*. Philos. Trans. R. Soc. Lond. B Biol. Sci. 314, 1–340 (1986). 22462104 10.1098/rstb.1986.0056

[27] P. D. Wes, C. I. Bargmann, *C. elegans* odour discrimination requires asymmetric diversity in olfactory neurons. Nature 410, 698–701 (2001). 11287957 10.1038/35070581

[28] J. T. Pierce-Shimomura, S. Faumont, M. R. Gaston, B. J. Pearson, S. R. Lockery, The homeobox gene lim-6 is required for distinct chemosensory representations in C. elegans. Nature 410, 694–698 (2001). 11287956 10.1038/35070575

[29] S. J. Cook, T. A. Jarrell, C. A. Brittin, Y. Wang, A. E. Bloniarz, M. A. Yakovlev, K. C. Q. Nguyen, L. T. Tang, E. A. Bayer, J. S. Duerr, H. E. Bülow, O. Hobert, D. H. Hall, S. W. Emmons, Whole-animal connectomes of both *Caenorhabditis elegans* sexes. Nature 571, 63–71 (2019). 31270481 10.1038/s41586-019-1352-7 PMC6889226

[30] A. Gordus, N. Pokala, S. Levy, S. W. Flavell, C. I. Bargmann, Feedback from network states generates variability in a probabilistic olfactory circuit. Cell 161, 215–227 (2015). 25772698 10.1016/j.cell.2015.02.018 PMC4821011

[31] G. P. Harris, V. M. Hapiak, R. T. Wragg, S. B. Miller, L. J. Hughes, R. J. Hobson, R. Steven, B. Bamber, R. W. Komuniecki, Three distinct amine receptors operating at different levels within the locomotory circuit are each essential for the serotonergic modulation of chemosensation in *Caenorhabditis elegans*. J. Neurosci. 29, 1446–1456 (2009). 19193891 10.1523/JNEUROSCI.4585-08.2009 PMC3418693

[32] L. Wang, H. Sato, Y. Satoh, M. Tomioka, H. Kunitomo, Y. Iino, A Gustatory Neural Circuit of *Caenorhabditis elegans* Generates Memory-Dependent Behaviors in Na(+) Chemotaxis. J. Neurosci. 37, 2097–2111 (2017). 28126744 10.1523/JNEUROSCI.1774-16.2017 PMC6705685

[33] B. F. Fosque, Y. Sun, H. Dana, C. T. Yang, T. Ohyama, M. R. Tadross, R. Patel, M. Zlatic, D. S. Kim, M. B. Ahrens, V. Jayaraman, L. L. Looger, E. R. Schreiter, Neural circuits. Labeling of active neural circuits in vivo with designed calcium integrators. Science 347, 755–760 (2015). 25678659 10.1126/science.1260922

[34] J. H. Chou, C. I. Bargmann, P. Sengupta, The *Caenorhabditis elegans odr-2* gene encodes a novel Ly-6-related protein required for olfaction. Genetics 157, 211–224 (2001). 11139503 10.1093/genetics/157.1.211 PMC1461492

[35] A. Fok, B. Brissette, T. Hallacy, H. Ahamed, E. Ho, S. Ramanathan, N. Ringstad, High-fidelity encoding of mechanostimuli by tactile food-sensing neurons requires an ensemble of ion channels. Cell Rep. 42, 112452 (2023). 37119137 10.1016/j.celrep.2023.112452 PMC10320741

[36] N. Pokala, Q. Liu, A. Gordus, C. I. Bargmann, Inducible and titratable silencing of *Caenorhabditis elegans* neurons in vivo with histamine-gated chloride channels. Proc. Natl. Acad. Sci. U.S.A. 111, 2770–2775 (2014). 24550306 10.1073/pnas.1400615111 PMC3932931

[37] J. M. Gray, J. J. Hill, C. I. Bargmann, A circuit for navigation in *Caenorhabditis elegans*. Proc. Natl. Acad. Sci. U.S.A. 102, 3184–3191 (2005). 15689400 10.1073/pnas.0409009101 PMC546636

[38] S. Hori, S. Oda, Y. Suehiro, Y. Iino, S. Mitani, OFF-responses of interneurons optimize avoidance behaviors depending on stimulus strength via electrical synapses. PLOS Genet. 14, e1007477 (2018). 29939997 10.1371/journal.pgen.1007477 PMC6034901

[39] Y. Iino, K. Yoshida, Parallel use of two behavioral mechanisms for chemotaxis in *Caenorhabditis elegans*. J. Neurosci. 29, 5370–5380 (2009). 19403805 10.1523/JNEUROSCI.3633-08.2009 PMC6665864

[40] J. T. Pierce-Shimomura, T. M. Morse, S. R. Lockery, The fundamental role of pirouettes in Caenorhabditis elegans chemotaxis. J. Neurosci. 19, 9557–9569 (1999). 10531458 10.1523/JNEUROSCI.19-21-09557.1999 PMC6782915

[41] J. C. Campbell, L. F. Polan-Couillard, I. D. Chin-Sang, W. G. Bendena, NPR-9, a galanin-like G-protein coupled receptor, and GLR-1 regulate interneuronal circuitry underlying multisensory integration of environmental cues in *Caenorhabditis elegans*. PLOS Genet. 12, e1006050 (2016). 27223098 10.1371/journal.pgen.1006050 PMC4880332

[42] W. Zou, J. Fu, H. Zhang, K. Du, W. Huang, J. Yu, S. Li, Y. Fan, H. A. Baylis, S. Gao, R. Xiao, W. Ji, L. Kang, T. Xu, Decoding the intensity of sensory input by two glutamate receptors in one C. elegans interneuron. Nat. Commun. 9, 4311 (2018). 30333484 10.1038/s41467-018-06819-5 PMC6193023

[43] D. R. Jones, Z. Wu, D. Chauhan, K. C. Anderson, J. Peng, A nano ultra-performance liquid chromatography-high resolution mass spectrometry approach for global metabolomic profiling and case study on drug-resistant multiple myeloma. Anal. Chem. 86, 3667–3675 (2014). 24611431 10.1021/ac500476a PMC6424491

[44] N. Seiler, [2] Liquid chromatographic methods for assaying polyamines using prechromatographic derivatization in Methods in Enzymology (Academic Press, 1983), vol. 94. p. 10–25. 10.1016/s0076-6879(83)94004-1 6621380

[45] G. Veeranagamallaiah, C. Sudhakar, Determination of polyamines by dansylation, benzoylation, and capillary electrophoresis. Methods Mol. Biol. 1631, 313–323 (2017). 28735407 10.1007/978-1-4939-7136-7_20

[46] D. A. Garsin, C. D. Sifri, E. Mylonakis, X. Qin, K. V. Singh, B. E. Murray, S. B. Calderwood, F. M. Ausubel, A simple model host for identifying Gram-positive virulence factors. Proc. Natl. Acad. Sci. U.S.A. 98, 10892–10897 (2001). 11535834 10.1073/pnas.191378698 PMC58570

[47] M. K. Chattopadhyay, C. W. Tabor, H. Tabor, Polyamines are not required for aerobic growth of *Escherichia coli*: Preparation of a strain with deletions in all of the genes for polyamine biosynthesis. J. Bacteriol. 191, 5549–5552 (2009). 19542271 10.1128/JB.00381-09 PMC2725612

[48] D. Manoel, M. Makhlouf, C. J. Arayata, A. Sathappan, S. Da'as, D. Abdelrahman, S. Selvaraj, R. Hasnah, J. D. Mainland, R. C. Gerkin, L. R. Saraiva, Deconstructing the mouse olfactory percept through an ethological atlas. Curr. Biol. 31, 2809–2818.e3 (2021). 33957076 10.1016/j.cub.2021.04.020 PMC8282700

[49] A. Hussain, L. R. Saraiva, D. M. Ferrero, G. Ahuja, V. S. Krishna, S. D. Liberles, S. I. Korsching, High-affinity olfactory receptor for the death-associated odor cadaverine. Proc. Natl. Acad. Sci. U.S.A. 110, 19579–19584 (2013). 24218586 10.1073/pnas.1318596110 PMC3845148

[50] A. Hussain, M. Zhang, H. K. Ucpunar, T. Svensson, E. Quillery, N. Gompel, R. Ignell, I. C. Grunwald Kadow, Ionotropic chemosensory receptors mediate the taste and smell of polyamines. PLOS Biol. 14, e1002454 (2016). 27145030 10.1371/journal.pbio.1002454 PMC4856413

[51] V. R. Heale, K. Petersen, C. H. Vanderwolf, Effect of colchicine-induced cell loss in the dentate gyrus and Ammon's horn on the olfactory control of feeding in rats. Brain Res. 712, 213–220 (1996). 8814895 10.1016/0006-8993(95)01416-0

[52] S. H. Rolen, P. W. Sorensen, D. Mattson, J. Caprio, Polyamines as olfactory stimuli in the goldfish *Carassius auratus*. J. Exp. Biol. 206, 1683–1696 (2003). 12682100 10.1242/jeb.00338

[53] R. R. Gainetdinov, M. C. Hoener, M. D. Berry, Trace amines and their receptors. Pharmacol. Rev. 70, 549–620 (2018). 29941461 10.1124/pr.117.015305

[54] I. Mori, Genetics of chemotaxis and thermotaxis in the *NematodeCaenorhabditis elegans*. Annu. Rev. Genet. 33, 399–422 (1999). 10690413 10.1146/annurev.genet.33.1.399

[55] D. A. Colosimo, J. A. Kohn, P. M. Luo, F. J. Piscotta, S. M. Han, A. J. Pickard, A. Rao, J. R. Cross, L. J. Cohen, S. F. Brady, Mapping interactions of microbial metabolites with human g-protein-coupled receptors. Cell Host Microbe 26, 273–282.e7 (2019). 31378678 10.1016/j.chom.2019.07.002 PMC6706627

[56] A. Nakamura, S. Kurihara, D. Takahashi, W. Ohashi, Y. Nakamura, S. Kimura, M. Onuki, A. Kume, Y. Sasazawa, Y. Furusawa, Y. Obata, S. Fukuda, S. Saiki, M. Matsumoto, K. Hase, Symbiotic polyamine metabolism regulates epithelial proliferation and macrophage differentiation in the colon. Nat. Commun. 12, 2105 (2021). 33833232 10.1038/s41467-021-22212-1 PMC8032791

[57] A. J. Michael, Polyamines in eukaryotes, bacteria, and archaea. J. Biol. Chem. 291, 14896–14903 (2016). 27268252 10.1074/jbc.R116.734780 PMC4946907

[58] S. Brenner, The genetics of *Caenorhabditis elegans*. Genetics 77, 71–94 (1974). 4366476 10.1093/genetics/77.1.71 PMC1213120

[59] E. Drenkard, B. G. Richter, S. Rozen, L. M. Stutius, N. A. Angell, M. Mindrinos, R. J. Cho, P. J. Oefner, R. W. Davis, F. M. Ausubel, A simple procedure for the analysis of single nucleotide polymorphisms facilitates map-based cloning in Arabidopsis. Plant Physiol. 124, 1483–1492 (2000). 11115864 10.1104/pp.124.4.1483 PMC1539302

[60] D. G. Gibson, L. Young, R. Y. Chuang, J. C. Venter, C. A. Hutchison III, H. O. Smith, Enzymatic assembly of DNA molecules up to several hundred kilobases. Nat. Methods 6, 343–345 (2009). 19363495 10.1038/nmeth.1318

[61] C. C. Mello, J. M. Kramer, D. Stinchcomb, V. Ambros, Efficient gene transfer in *C. elegans*: Extrachromosomal maintenance and integration of transforming sequences. EMBO J. 10, 3959–3970 (1991). 1935914 10.1002/j.1460-2075.1991.tb04966.x PMC453137

[62] J. Schindelin, I. Arganda-Carreras, E. Frise, V. Kaynig, M. Longair, T. Pietzsch, S. Preibisch, C. Rueden, S. Saalfeld, B. Schmid, J. Y. Tinevez, D. J. White, V. Hartenstein, K. Eliceiri, P. Tomancak, A. Cardona, Fiji: An open-source platform for biological-image analysis. Nat. Methods 9, 676–682 (2012). 22743772 10.1038/nmeth.2019 PMC3855844

[63] Y. Simon-Manso, M. S. Lowenthal, L. E. Kilpatrick, M. L. Sampson, K. H. Telu, P. A. Rudnick, W. G. Mallard, D. W. Bearden, T. B. Schock, D. V. Tchekhovskoi, N. Blonder, X. Yan, Y. Liang, Y. Zheng, W. E. Wallace, P. Neta, K. W. Phinney, A. T. Remaley, S. E. Stein, Metabolite profiling of a NIST standard reference material for human plasma (SRM 1950): GC-MS, LC-MS, NMR, and clinical laboratory analyses, libraries, and web-based resources. Anal. Chem. 85, 11725–11731 (2013). 24147600 10.1021/ac402503m

[64] C. A. Smith, G. O'Maille, E. J. Want, C. Qin, S. A. Trauger, T. R. Brandon, D. E. Custodio, R. Abagyan, G. Siuzdak, METLIN: A metabolite mass spectral database. Ther. Drug Monit. 27, 747–751 (2005). 16404815 10.1097/01.ftd.0000179845.53213.39

