# Supplementary material for: Chemosensory detection of polyamine metabolites guides C. elegans to nutritive microbes
Source: Sci Adv. 2024 Mar 22;10(12):eadj4387. doi: 10.1126/sciadv.adj4387 (PMC10959419; doi:10.1126/sciadv.adj4387)
Supplement: Supplementary file 1 — Figs. S1 to S11 Legends for tables S1 and S2 Tables S3 to S5 Legends for data files S1 to S7 [file sciadv.adj4387_sm.pdf]

Science Advances
AAAS

Supplementary Materials for

Chemosensory detection of polyamine metabolites guides *C. elegans* to nutritive microbes

Benjamin Brissette *et al.*

Corresponding author: Niels Ringstad, Niels.Ringstad@med.nyu.edu

Sci. Adv. **10**, eadj4387 (2024)
DOI: 10.1126/sciadv.adj4387

The PDF file includes:

Figs. S1 to S11
Legends for tables S1 and S2
Tables S3 to S5
Legends for data files S1 to S7

Other Supplementary Material for this manuscript includes the following:

Tables S1 and S2
Data files S1 to S7

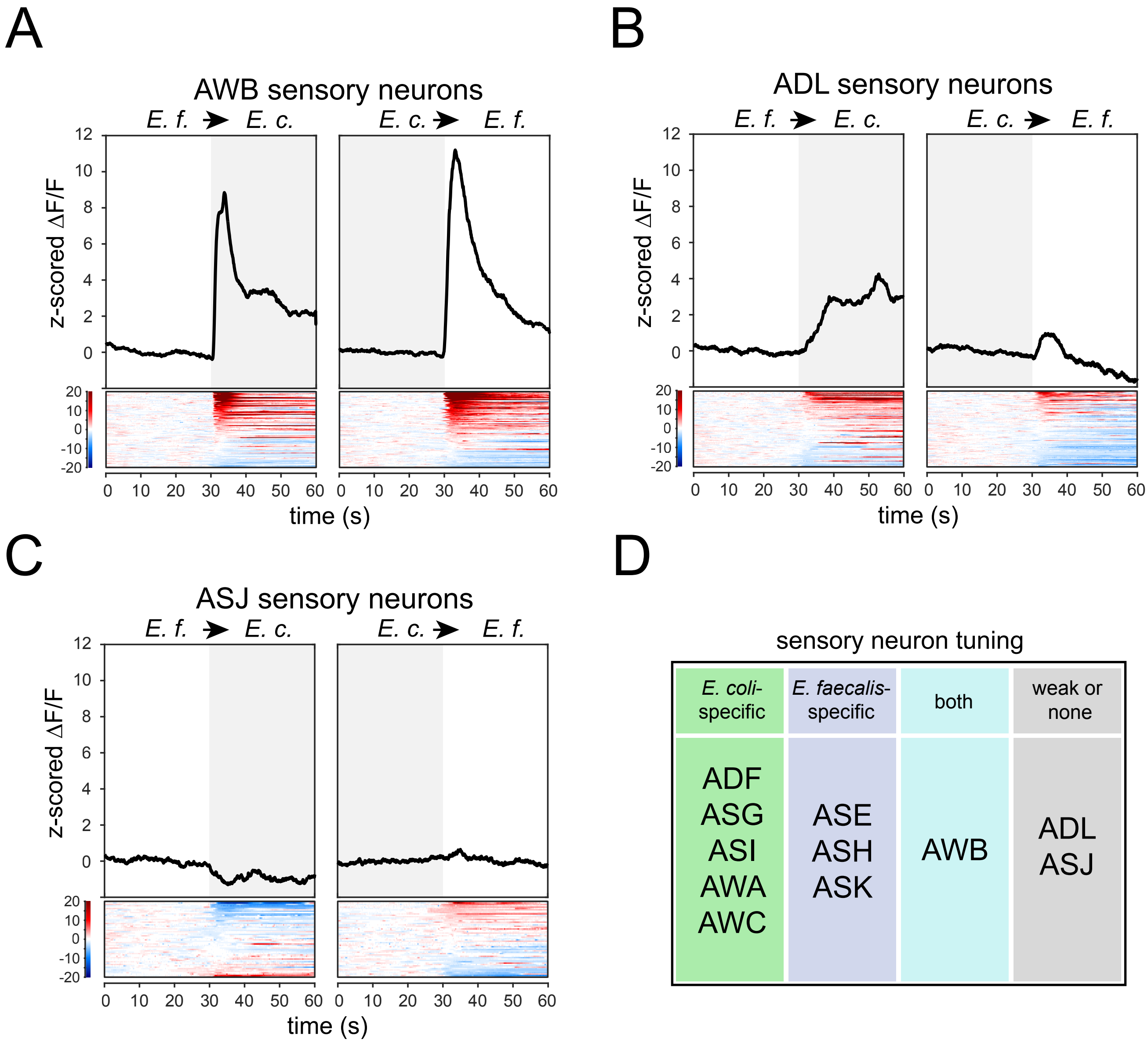

Fig. S1. Responses of C. elegans chemosensory neurons to microbe- conditioned media.
(A-C) Z-scored calcium signals in AWB, ADF, and ADL neurons in response to switches between *E. faecalis*-conditioned and *E. coli*-conditioned media. Data are plotted from 95 trials of AWBs, 96 trials of ADLs, and 56 trials of ASJs.
(D) Table categorizing tuning of recorded neurons to *E. coli* and *E. faecalis*.
In A-C traces of the mean Z-scored signals are shown above heat maps showing the Z-scored signals from individual trials.

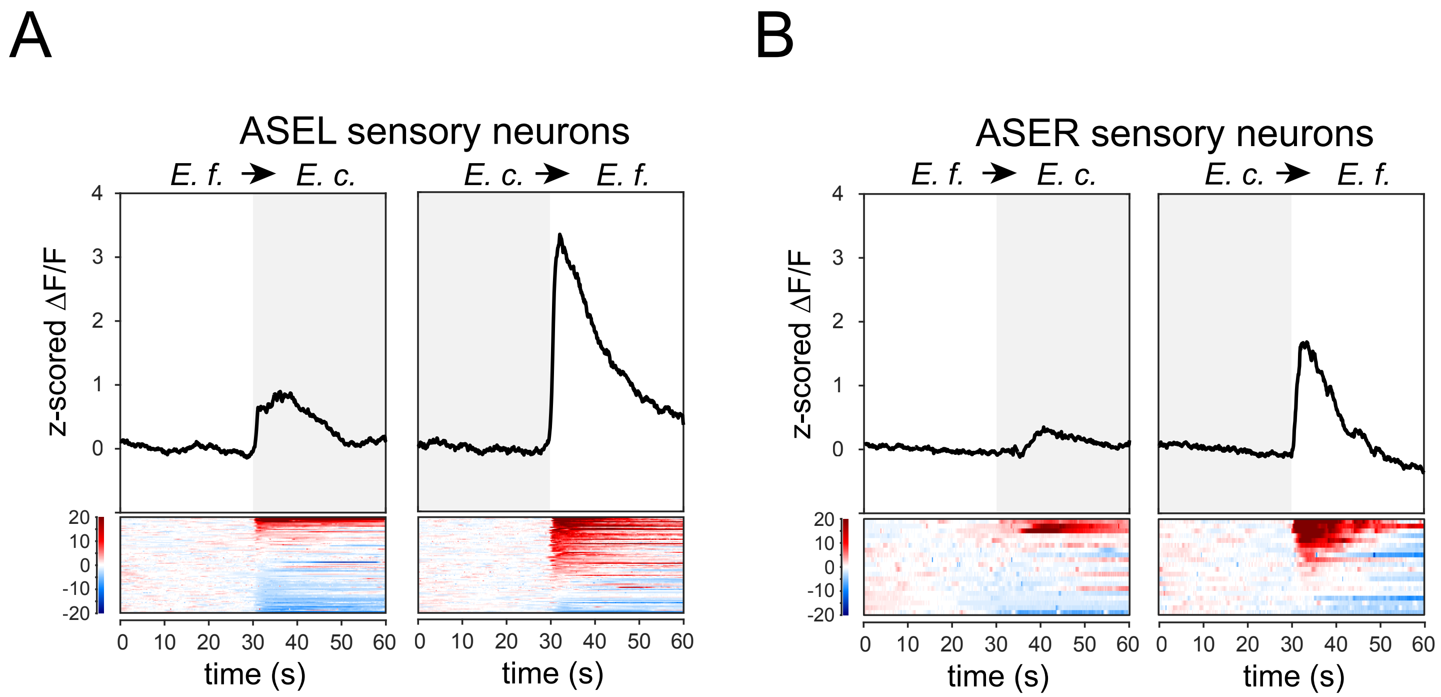

Fig. S2. ASEL and ASER neurons respond similarly to microbe- conditioned media.

Data from Fig. 2D are replotted according to laterality of ASE neurons.

(A-B) Z-scored calcium signals in ASEL and ASER neurons in response to switches between *E. faecalis*-conditioned and *E. coli*-conditioned media. Data are plotted from 88 trials of ASELs and 20 trials of ASERs.

Traces of the mean Z-scored signals are shown above heat maps showing the Z-scored signals from individual trials.

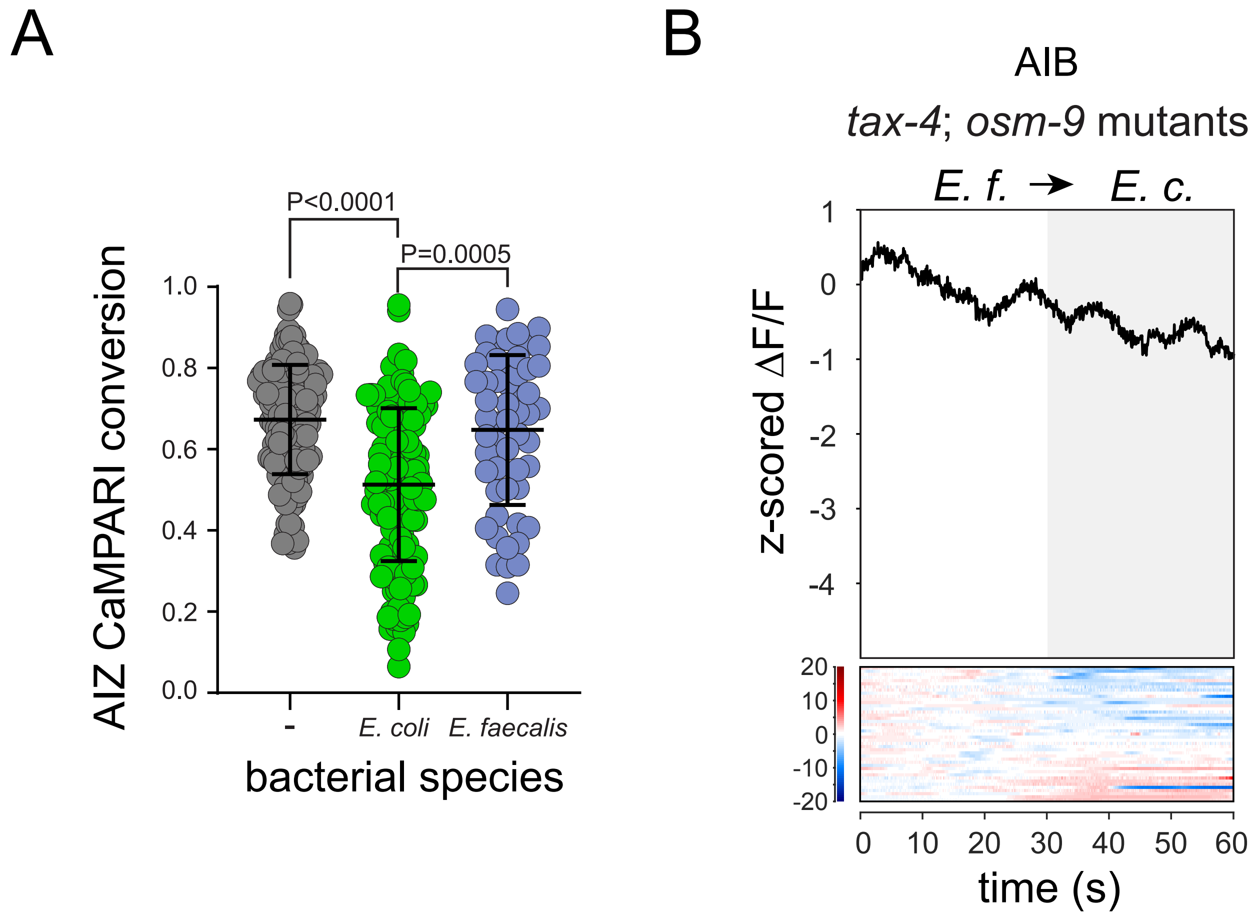

Fig. S3. AIZ interneurons are regulated by *E. coli*-sensing neurons and AIB microbe-responses require TAX-4 and OSM-9 chemotransduction channels.
(A) CaMPARI signals in control animals (n = 151 cells) and animals exposed either to nutritive *E. coli* (n = 171 cells) or pathogenic *E. faecalis* (n = 54 cells). Error bars show mean plus or minus standard deviation. P values were computed using the Kolmogorov-Smirnov test and corrected for multiple comparisons. Each point represents one cell.
(B) Calcium responses of AIB neurons in tax-4; osm-9 double-mutant animals to *E. coli*-conditioned media after adaptation to *E. faecalis*-conditioned media (n = 43 trials). Traces of the mean Z-scored signals from AIBs are shown above heat maps showing the Z-scored signals from individual trials.

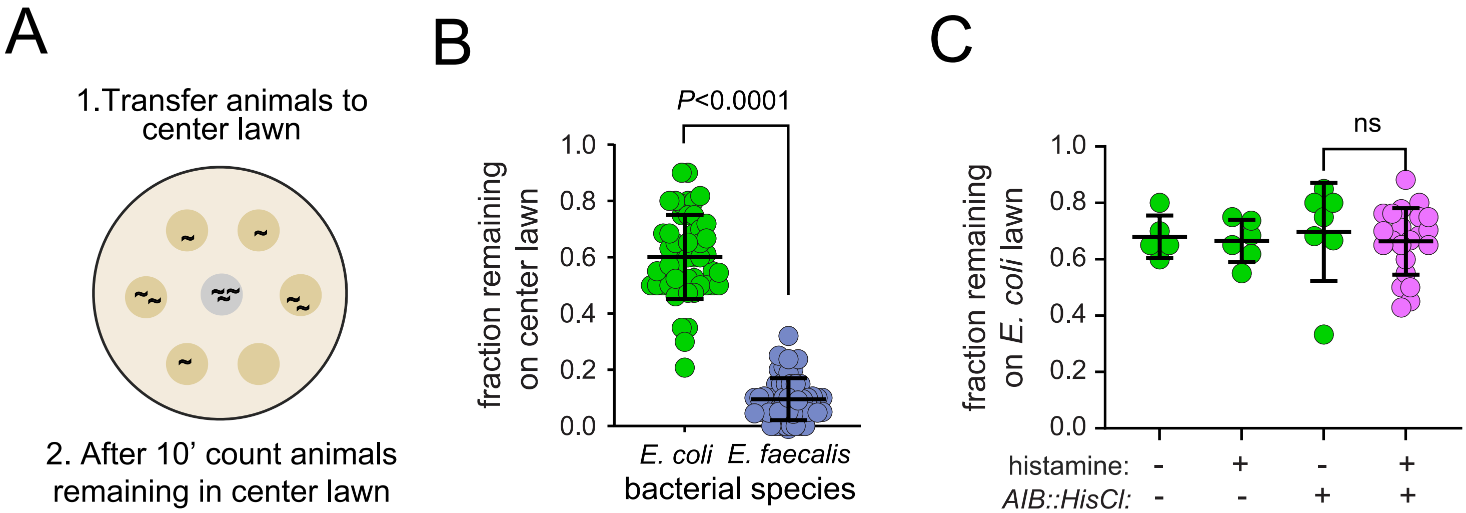

Fig. S4. Dwelling on patches of bacteria does not require AIB neurons.
(A) Schematic of an assay for retention of animals by lawns of bacteria.
(B) Fraction of animals retained by bacteria computed from behavioral responses of *C. elegans* to patches of *E. coli* and *E. faecalis* (n = 50 trials for *E. coli* and 52 trials for *E. faecalis*). Student's t-test was used to compute the indicated P value. Each point represents one trial.
(C) Retention by lawns of *E. coli* of the wild type or transgenic animals expressing HisCl in the AIB neurons in the presence or absence of histamine. In the presence of histamine, AIB neurons of transgenics are silenced. 5 trials of the wild type were performed without and 6 with histamine. 7 trials of transgenics were performed without and 22 with histamine. Each trial used 20 animals. Error bars represent mean plus or minus standard deviation. Student's t-test was used to compute the indicated P value.

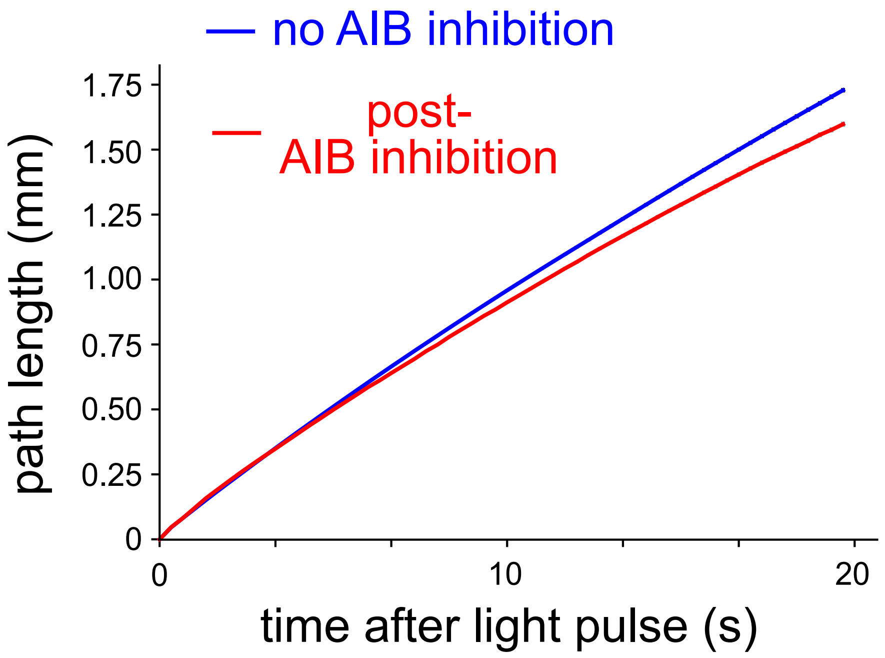

Fig. S5. Inhibition of AIB interneurons has little effect on speed during foraging. Distance traveled by animals in total after receiving a pulse of AIB inhibition (red) or not (blue).

Error bars show standard error of the mean.

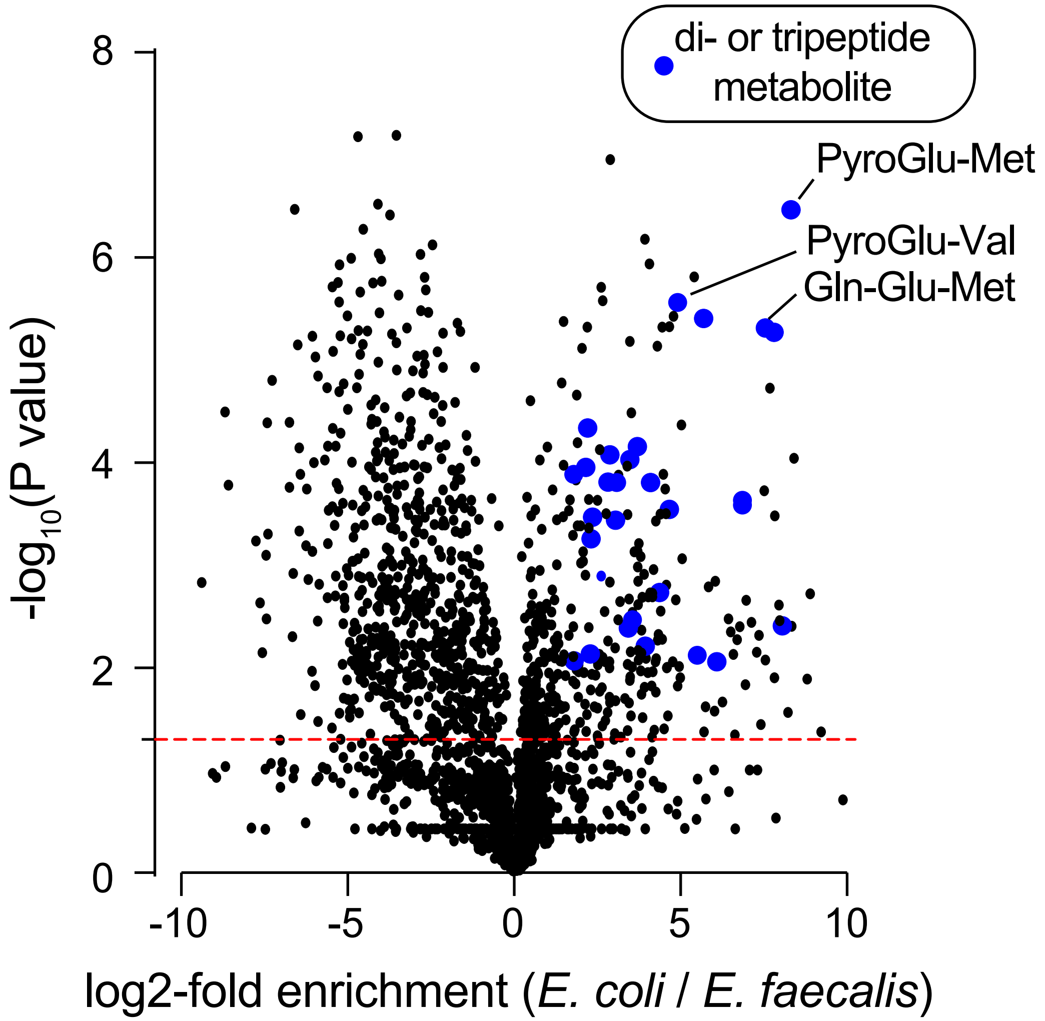

Fig. S6. Di- and tripeptides are enriched in nutritive *E. coli*.
Volcano plot comparing *E. coli*- and *E. faecalis*-conditioned media. Log2-Fold-enrichment in *E. coli* over *E. faecalis* is plotted on the horizontal axis, and the associated P value is plotted on the vertical axis. The red dashed line shows P = 0.05. Di- and tripeptide metabolites are highlighted in blue.

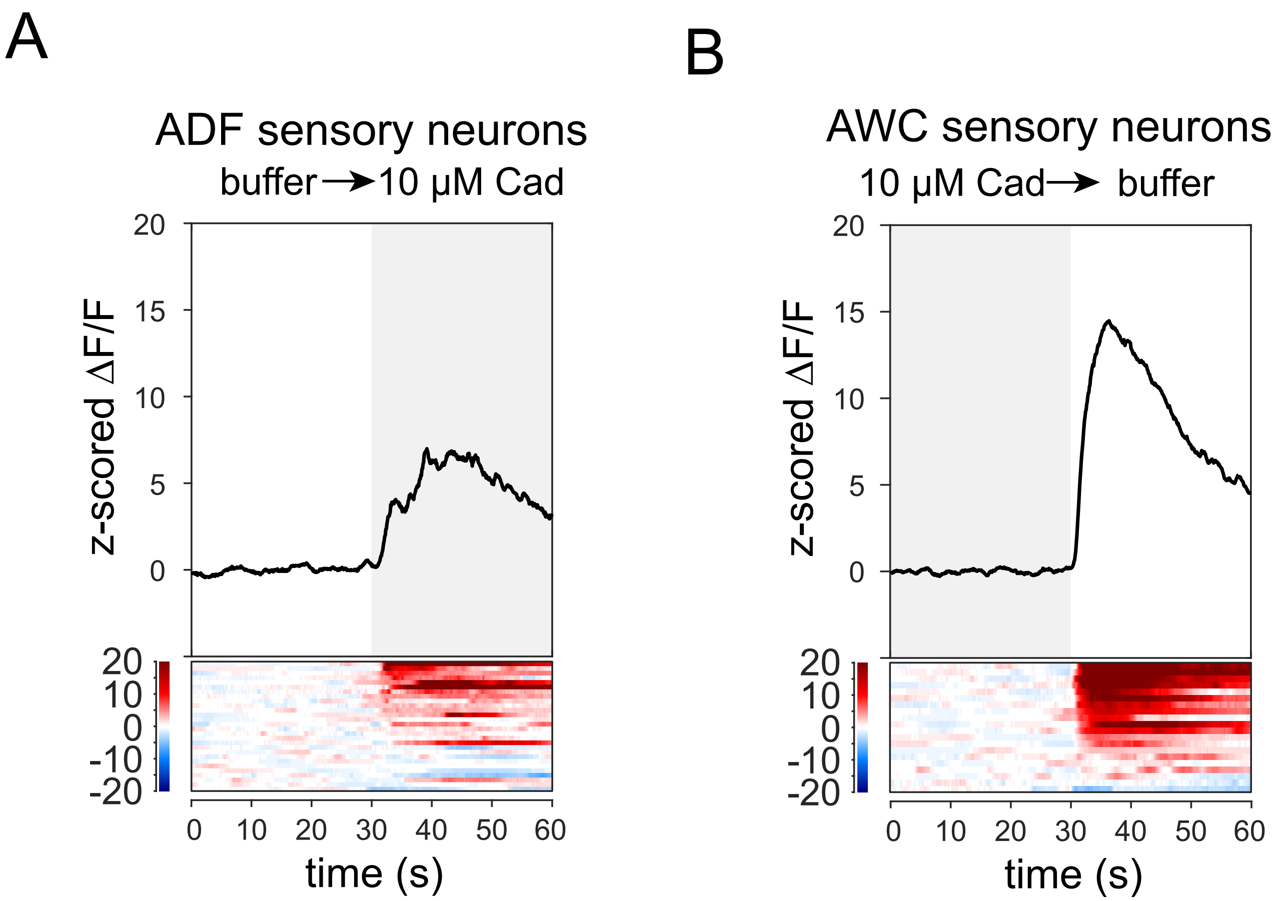

Figure S7. Responses of ADF and AWC neurons to 10 μM cadaverine.
(A-B) Responses of ADF and AWC neurons to 10 μM cadaverine. Data are plotted from 28 trials of ADFs and 20 trials of AWCs.
Traces of the mean Z-scored signals are shown above heat maps showing the Z-scored signals from individual trials.

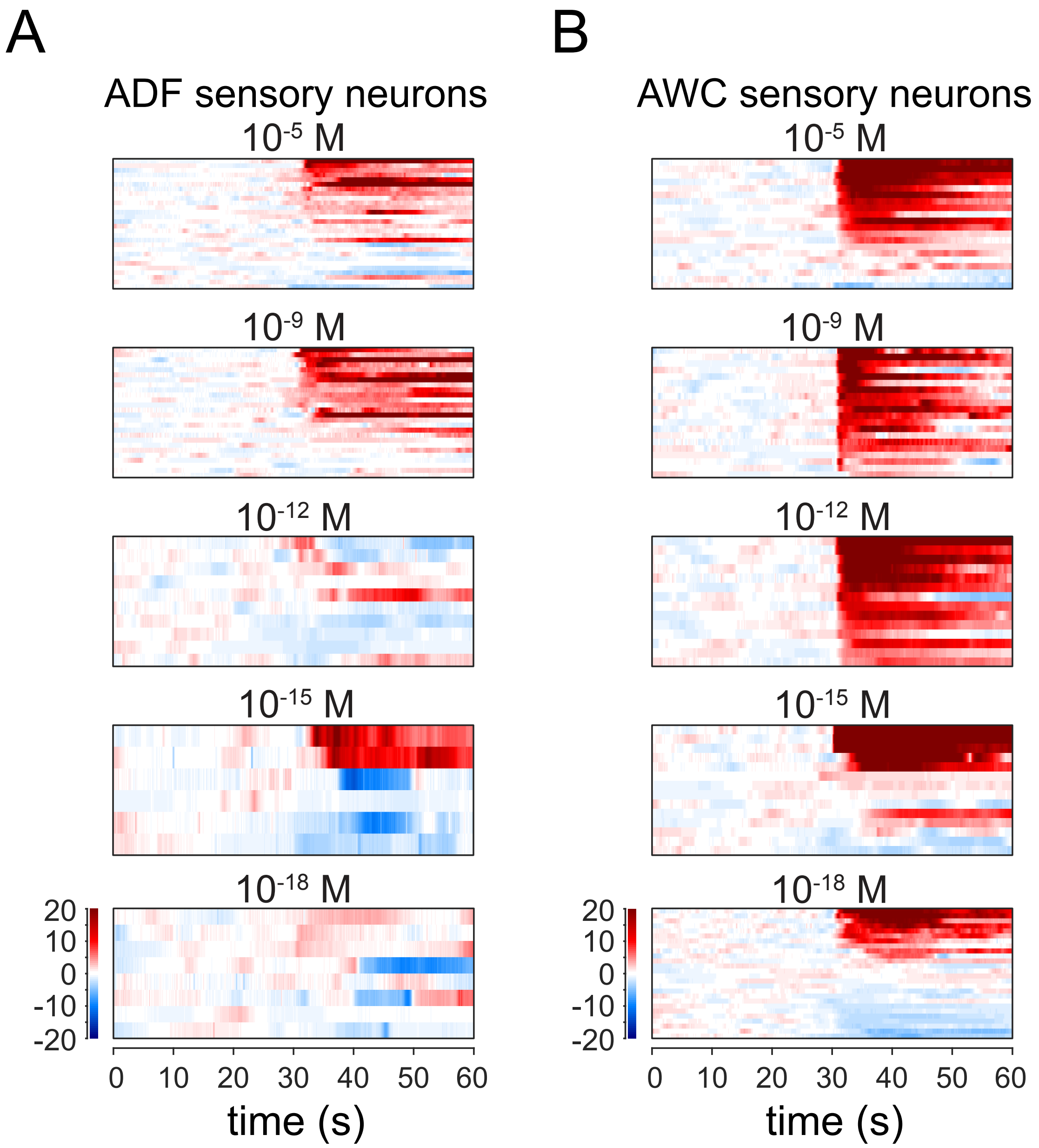

Fig. S8. Responses of ADF and AWC neurons to decreasing concentrations of cadaverine.

(A-B) Top row: responses of ADF and AWC neurons to 10 μM (10^{-5}M) cadaverine. Data are from 28 trials of ADFs and 20 trials of AWCs.

Second row: responses to 1nM (10^{-9}M) cadaverine. Data are from 26 trials of ADFs and 20 trials of AWCs.

Third row: responses to 1pM (10^{-12}M) cadaverine. Data are from 10 trials of ADFs and 14 trials of AWCs.

Fourth row: responses to 1fM (10^{-15}M) cadaverine. Data are from 6 trials of ADFs and 14 trials of AWCs.

Fifth row: responses to 10^{-18}M cadaverine. Data are from 8 trials of ADFs and 26 trials of AWCs.

Shown are heat maps showing the Z-scored signals from individual trials.

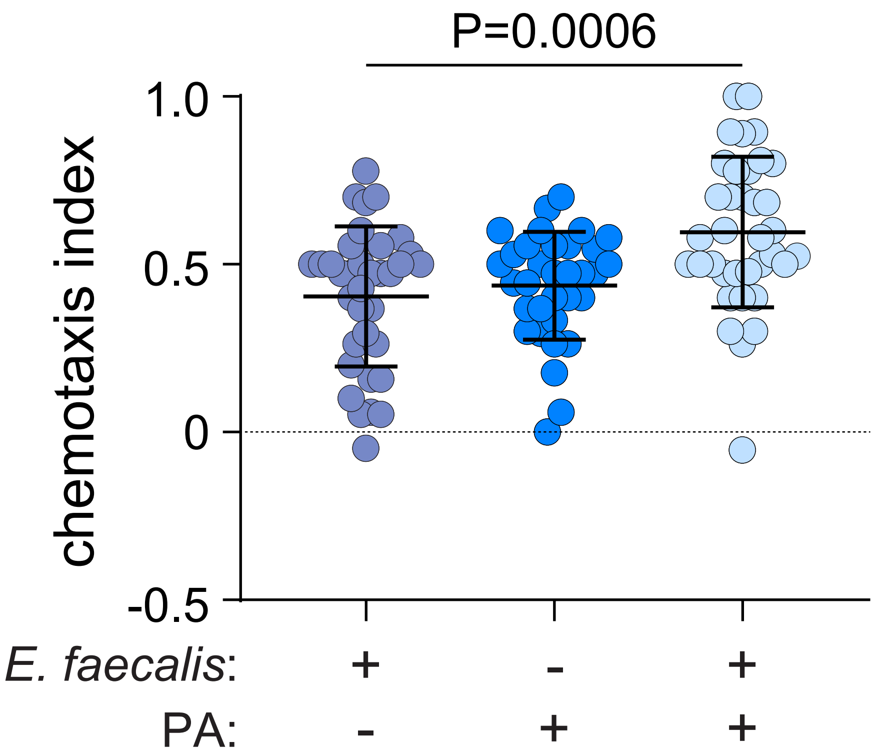

Fig. S9. Polyamines increase attraction to *E. faecalis*.
Chemotaxis indices computed from behavioral responses of *C. elegans* to *E. faecalis*-conditioned media (n=35 trials), polyamine odorants (n=33 trials), or polyamine odorants added to *E. faecalis*-conditioned media (n = 36 trials). Each trial used 20 animals. Student's t-test was used to compute the indicated P value. Error bars represent mean plus or minus standard deviation. Each point represents one trial.

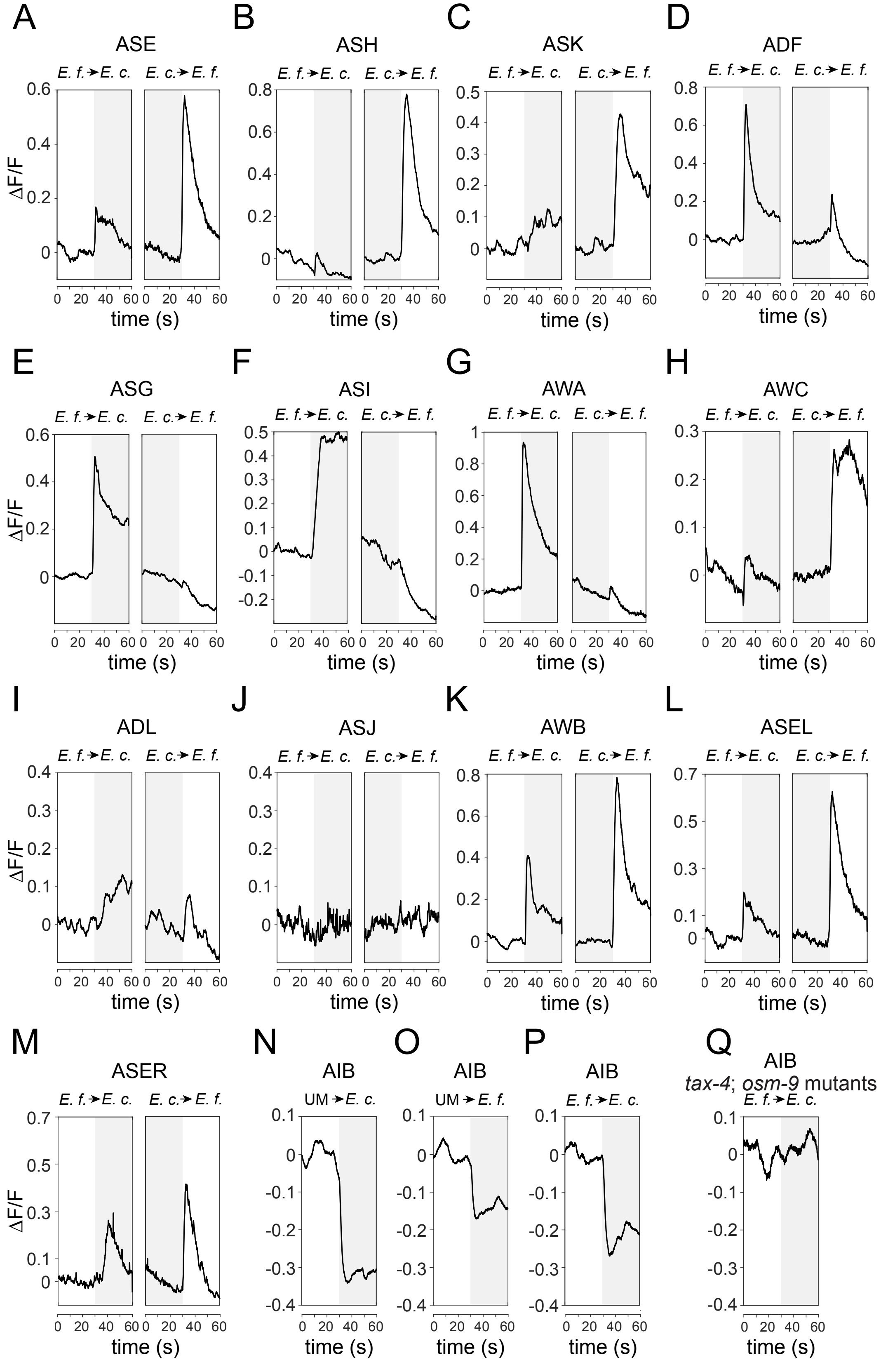

Fig. S10. Non-Z-scored GCaMP6f responses of *C. elegans* neurons to microbe-conditioned media.
Plots show the mean ΔF/F of all trials of neurons responding to the indicated stimuli.
(A-K) Corresponding to Fig. 1 and Fig. S1. Data are plotted from 108 trials of ASEs, 117 trials of ASHs, 105 trials of ASKs, 70 trials of ADFs, 92 trials of ASGs, 86 trials of ASIs, 110 trials of AWAs, 116 trials of AWCs, 96 trials of ADLs, 56 trials of ASJs, and 95 trials of AWBs.
(L-M) Corresponding to Fig. S2. Data are plotted from 88 trials of ASELs and 20 trials of ASERs.
(N-P) Corresponding to Fig. 2. Data are plotted from 33 trials of *E. coli*-conditioned media, 60 trials of *E. faecalis*-conditioned media, and 70 trials of *E. coli*-conditioned media after adaptation to *E. faecalis*-conditioned media.
(Q) Corresponding to Fig. S3. Data are plotted from 43 trials of *tax-4*; *osm-9* double-mutant animals to *E. coli*-conditioned media after adaptation to *E. faecalis*-conditioned media.

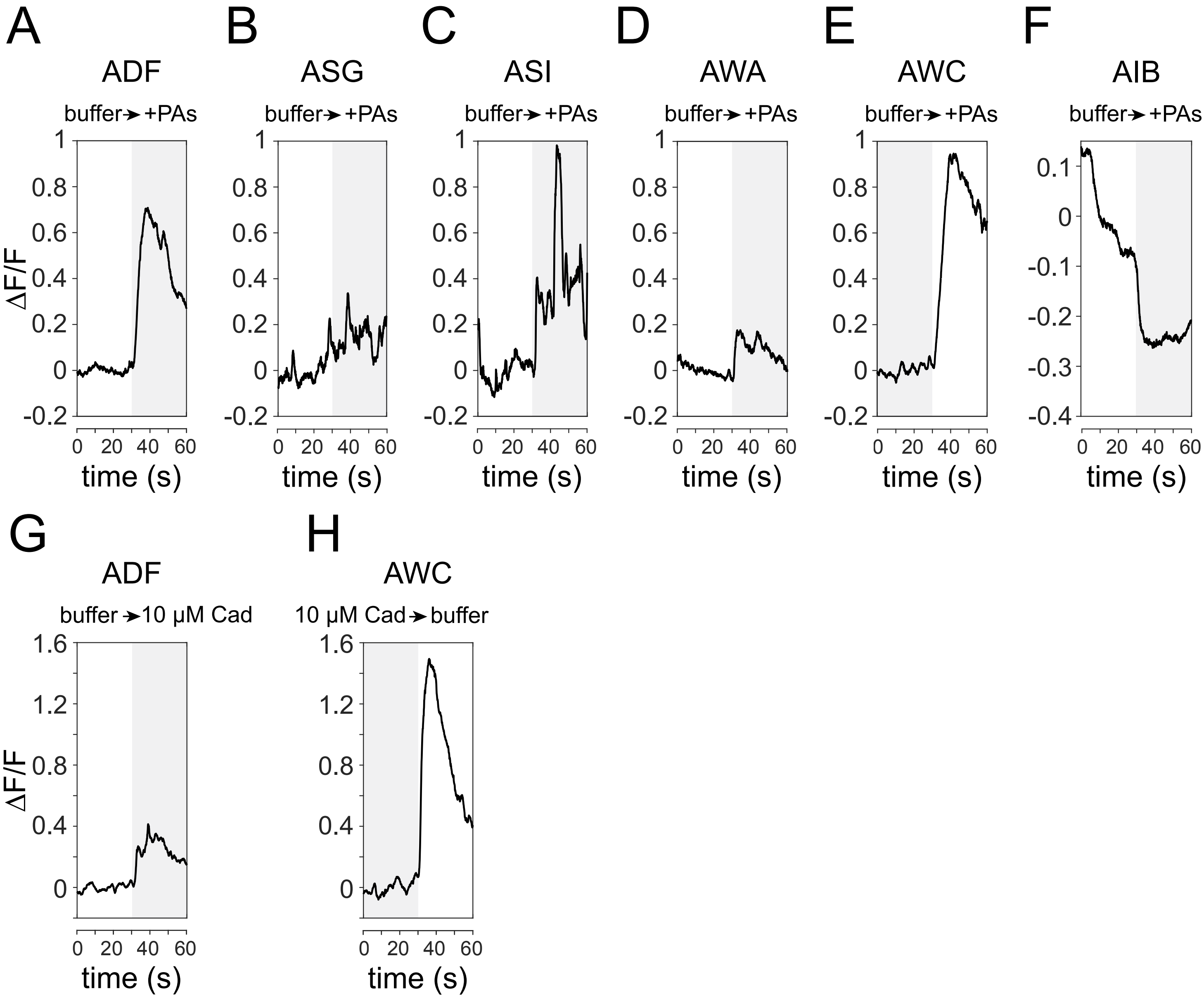

Fig. S11. Non-Z-scored GCaMP6f responses of *C. elegans* neurons to polyamine stimuli.
Plots show the mean ΔF/F of all trials of neurons responding to the indicated stimuli.
(A-F) Corresponding to Fig. 5. Data are plotted from 18 trials of ADFs, 8 trials of ASGs, 6 trials of ASIs, 20 trials of AWAs, 22 trials of AWCs, and 50 trials of AIBs.
(G-H) Corresponding to Fig. S7. Data are plotted from 28 trials of ADFs and 20 trials of AWCs.

Table S1. Metabolites differentially expressed by *E. coli* and *E. faecalis*.

This table contains data regarding Fig. 3. Metabolite identity is determined as noted in Methods, above. Enrichment values across each of the three replicates for *E. coli*-conditioned media, *E. faecalis*-conditioned media, and Luria Broth (unconditioned media) are shown.
Table is included in a separate file due to size.

Table S2. Targeted polyamine profiling of a panel of microbes.

This table contains data regarding Figs. 3 and 4. Metabolite identity is determined as noted in Methods, above. Enrichment values are shown for all replicates of each microbe. 9 replicates were performed for *E. coli*-conditioned media, *E. faecalis*-conditioned media, and Luria Broth and 3 replicates were performed for all other samples. Highlighted values indicate undetected values that were replaced with the detection threshold.
Table is included in a separate file due to size.

Genotype	Source	Strain Name
kyEx4018 [*Pinx-1::GCAMP3* 50ng/uL, *unc-122::dsRed* 10ng/uL]	(*30*)	CX13440
kyIs620 [pNP472 (*inx-1::HisCl1::SL2::GFP*), 30 ng/uL + *myo-3::mCherry*, 5 ng/uL]	(*36*)	CX15457
tax-4(p678) III; *osm-9*(ky10) IV; *him-5*(e1490) V	(*26*)	EG4767
wzIs195[*Podr-2b::CaMPARI*]; *lite-1*(ce314) X	This study	FQ2183
wzIs251[*parl3::GCaMP6F punc122::gfp*]; *lite-1*(ce314) X.	This study	FQ2537
tax-4(p678); *osm-9*(ky10); kyEx4018[*Pinx-1::GCamp3*]	This study	FQ2719
wzEx663[*pinx-1::arch-rfp punc122::gfp*]; *lite-1*(ce314) X.	This study	FQ2732
wild-type	Caenorhabditis Genetics Center	N2 Bristol

Table S3. Strains used in this study

This table contains a list of the strains of *C. elegans* that were used in this study, as well as their origins.

Primer Name	Sequence (5' - 3')
lite-1 WT SNAP forward primer	CCATTTTCTGTGTTCTTCTGGATTCTTATGCTG
lite-1 WT SNAP reverse primer	ACTGGGCACTCATTCCGACAAACATC
lite-1(ce314) SNAP forward primer	AACCATTTTCTGTGTTCTTCTGGATTCTTATGCTA
lite-1(ce314) SNAP reverse primer	GCAAACACCATATGAGTAGAACAAAACAAACGA
tax-4 WT SNAP forward primer	GCCATCGGAAGATGCCGGTTG
tax-4(p678) SNAP forward primer	CGCCATCGGAAGATGCCGGATA
tax-4 SNAP universal reverse primer	GCATCAGCGGCAACGAAGTTCAGTAT
osm-9 WT SNAP forward primer	AATGGCTAGGTGGAGGGCTGATCG
osm-9(ky10) SNAP forward primer	TGTACAATGGCTAGGTGGAGGGCTGATAA
osm-9 SNAP universal reverse primer	CTCTCAAACTTCTAAATCTTCCAGAACGTGCA

Table S4. Genotyping primers

This table contains information on the oligonucleotide primers used to verify genotypes of relevant *C. elegans* strains.

1. Constructed Plasmids	
Plasmid name	Description
pBB8	$p_{odr\text{-}2b}$::*CaMPARI*
pBB20	$p_{arl\text{-}3}$::*GCaMP6f*
pAEF1	$p_{\text{inx-1}}$::*Arch::RFP*
2. Plasmid concentrations used to generate extrachromosomal arrays	
Construct name	*Injection concentration (ng/µL)*
$p_{odr\text{-}2b}$::*CaMPARI*	50
$p_{arl\text{-}3}$::*GCaMP6f*	50
$p_{\text{inx-1}}$::*Arch::RFP*	40
$p_{unc\text{-}122}$::*GFP*	50-65

Table S5. Plasmids for transgenesis

This table contains information on the plasmids used to generate some of the transgenic *C. elegans* strains used.

CAPTIONS FOR SOURCE DATA FILES

Data S1. Source data for Figure 1

Data set includes: instantaneous and end-point lawn occupancy data plotted in Figures 1A-B, a maximum projection image of a volume acquired from the *C. elegans* strain used for calcium imaging of chemosensory neurons shown in Figure 1C, and spreadsheets containing background corrected calcium measurements shown in Figures 1D-K.

These and all other spreadsheets containing imaging data (.xlsx files) follow the same format. Data are background subtracted fluorescence values extracted from a labeled ROI corresponding to the cell indicated in the file name. Each row is a trial, each column is a frame. Each trial is organized such that it contains 30 seconds prior to the transition described in the file name and 30 seconds following that transition.

Data S2. Source data for Figure 2

Data set includes: A maximum projection image of CaMPARI fluorescence in the head of a *C. elegans* strain used for CaMPARI experiments shown in Figure 2B, source data for CaMPARI measurements plotted in Figure 2C, Excel spreadsheets containing background corrected calcium measurements shown in Figures 2D-F, source data for chemotaxis measurements shown in Figures 2H-I, and source data for effect of AIB silencing on locomotion shown in Figure 2K.

Data S3. Source data for Figure 3

Data set includes: source data for the PCA and volcano plots shown in Figures 3A-B, source data for m/z plots shown in Figures C-E, source data for plots showing retention profiles of dansylated polyamines shown in Figure 3F, and source data for enrichment of polyamine species shown in Figure 3G.

Data S4. Source data for Figure 4

Data set includes: source data for plot of CaMPARI signals shown in Figure 4A and source data for enrichment of cadaverine in media conditioned by different microbes shown in Figure 4B.

Data S5. Source data for Figure 5

Data set includes: Spreadsheets (.xlsx files) containing source data for plots of calcium signals in sensory neurons shown in Figures 5A-H, and measurements of chemotaxis behavior plotted in Figure 5I.

Data S6. Source data for Figure 6

Data set includes: source data for enrichment of polyamine species in wild-type vs. polyamine-synthesis-deficient *E. coli* plotted in Figure 6A and source data for measurements of chemotaxis behavior plotted in Figure 6B.

Data S7. Source data for Supplementary Figures

Figure S1 Data

Data set includes source data for plots of calcium signaling in sensory neurons shown in Figure S1A-C.

Figure S2 Data

Data set includes source data for plots of calcium signaling in sensory neurons shown in Figure S2A-B.

Figure S3 Data

Data set includes: measurements of CaMPARI signals plotted in Figure S3A and a spreadsheet containing source data for calcium measurements plotted in Figure S3B.

Figure S4 Data

Data set includes: source data for behavioral measurements plotted in Figures S4B-C.

Figure S5 Data

Data set includes source data for measurements of the effect of optogenetic inhibition of AIBs on speed plotted in Figure S5.

Figure S6 Data

Data set includes source data for the volcano plot shown in Figure S6.

Figure S7 Data

Data set includes source data for plots of calcium signaling in sensory neurons shown in Figure S7A-B.

Figure S8 Data

Data set includes source data for heatmaps of calcium signaling in sensory neurons shown in Figure S8A-B.

Figure S9 Data

Data set includes source data for measurements of chemotaxis behavior plotted in Figure S9.
